# Proteasome Assembly Chaperone 3 Defines Metabolic-Immune Programs and Poor Prognosis in Breast Cancer via Multi-Omics Approaches

**DOI:** 10.7150/jca.126116

**Published:** 2026-02-18

**Authors:** Sachin Kumar, Hoang Dang Khoa Ta, Hao-Chien Yang, Chia-Lung Shih, Dahlak Daniel Solomon, Ching-Chung Ko, Do Thi Minh Xuan, Yung-Kuo Lee, Kai-Fu Chang, Hui-Ru Lin, Shu-Huei Kao, Jian-Ying Chuang, Jian-Bin Chen, Chih-Yang Wang, Ngoc Uyen Nhi Nguyen

**Affiliations:** 1 PhD Program for Cancer Molecular Biology and Drug Discovery, College of Medical Science and Technology, Taipei Medical University, Taipei 11031, Taiwan.; 2 Graduate Institute of Cancer Biology and Drug Discovery, College of Medical Science and Technology, Taipei Medical University, Taipei 11031, Taiwan.; 3 Faculty of Applied Sciences and Biotechnology, Shoolini University of Biotechnology and Management Sciences Himachal Pradesh 173229, India.; 4 School of Computer Science, Duy Tan University, Da Nang, Vietnam.; 5 DTU AI and Data Science Hub (DAIDASH), Duy Tan University, Da Nang, Vietnam.; 6 Department of Surgery, Division of Neurosurgery, Shuang Ho Hospital, Taipei Medical University New Taipei City 23561, Taiwan.; 7 Ph.D. Program in Medical Biotechnology, College of Medical Science and Technology, Taipei Medical University, Taipei, Taiwan.; 8 Clinical Research Center, Ditmanson Medical Foundation Chiayi Christian Hospital, Chiayi City 60002, Taiwan.; 9 Yogananda School of AI Computers and Data Sciences, Shoolini University Solan 173229, India.; 10 Department of Medical Imaging, Chi-Mei Medical Center, Tainan 710402, Taiwan.; 11 Department of Health and Nutrition, Chia Nan University of Pharmacy and Science, Tainan 71710, Taiwan.; 12 School of Medicine, College of Medicine, National Sun Yat-Sen University, Kaohsiung 80424, Taiwan.; 13 Van Lang University, 69/68 Dang Thuy Tram Street, Binh Loi Trung Ward, Ho Chi Minh City, Vietnam.; 14 School of Medicine, National Defense Medical University, Taipei 11490, Taiwan.; 15 Medical Laboratory, Medical Education and Research Center, Kaohsiung Armed Forces General Hospital, National Defense Medical University, Kaohsiung 80284, Taiwan.; 16 Division of Experimental Surgery Center, Department of Surgery, Tri-Service General Hospital, National Defense Medical University, Taipei 11490, Taiwan.; 17 Institute of Medical Science and Technology, National Sun Yat-Sen University, Kaohsiung 80424, Taiwan.; 18 Nursing Department, Kaohsiung Armed Forces General Hospital, National Defense Medical University, Kaohsiung 80284, Taiwan.; 19 School of Medical Laboratory Science and Biotechnology, College of Medical Science and Technology, Taipei Medical University, Taipei, Taiwan.; 20 The Ph.D. Program for Neural Regenerative Medicine, Taipei Medical University, Taipei 11031, Taiwan.; 21 Research Center for Neuroscience, Taipei Medical University, Taipei, Taiwan.; 22 TMU Research Center of Cancer Translational Medicine, Taipei, Taiwan.; 23 International Master Program in Medical Neuroscience, College of Medical Science and Technology, Taipei Medical University, Taipei, Taiwan.; 24 Ph.D. Program in Drug Discovery and Development Industry, College of Pharmacy, Taipei Medical University, Taipei, Taiwan.; 25 Department of Biomedical Science and Environmental Biology, Kaohsiung Medical University, Kaohsiung 80708, Taiwan.; 26 Department of Radiology, Ditmanson Medical Foundation Chiayi Christian Hospital, Chiayi, Taiwan.; 27 Center for Regenerative Medicine, University of South Florida Health Heart Institute, Tampa, FL 33602, U.S.A.; 28 Division of Cardiology, Department of Internal Medicine, Morsani School of Medicine, University of South Florida, Tampa, FL 33602, U.S.A.

**Keywords:** PSMG3 (proteasome assembly chaperone 3), hypoxia, fatty acid metabolism, immune infiltration, tumor microenvironment, single-cell sequencing, breast cancer

## Abstract

The proteasome assembly chaperone (*PSMG*) gene family (comprised of *PSMG1*, *PSMG2*, *PSMG3*, and *PSMG4*) plays a critical role in proteasome biogenesis; however, its involvement in breast cancer remains poorly understood. Among these chaperones, *PSMG3* is uniquely and markedly elevated in breast cancer and is associated with poor clinical outcomes. We systematically investigated the roles of *PSMG* family genes in breast cancer by integrating multi-cohort genomic and transcriptomic datasets, including TCGA-BRCA, METABRIC, and multiple NCBI GEO cohorts. Comprehensive bioinformatics analyses were performed using bulk RNA sequencing and single-cell RNA sequencing data. A gene set enrichment analysis (GSEA) and immune infiltration analyses (CIBERSORT and TIMER) were applied to characterize dysregulated biological pathways, tumor microenvironmental features, and clinical relevance. In addition, molecular docking analyses were conducted to assess the druggability and binding potential of PSMG family proteins with selected small-molecule inhibitors. Elevated PSMG3 expression was consistently associated with poor survival outcomes across multiple breast cancer cohorts. Functional enrichment analyses revealed that PSMG3-high tumors were characterized by activation of hypoxia-related signaling pathways and dysregulated fatty acid metabolism, suggesting a role for PSMG3 in metabolic reprogramming. Immune deconvolution analyses further demonstrated significant correlations between PSMG3 expression and distinct immune cell populations within the tumor microenvironment. These findings were supported by single-cell transcriptomic profiling, which revealed subtype-specific expression patterns of PSMG3 in malignant epithelial cell populations. This integrative multi-omics analysis identified PSMG3 as a clinically relevant proteasome assembly chaperone associated with aggressive breast cancer phenotypes, metabolic dysregulation, and tumor immune contexture. Collectively, these results highlight PSMG3 as a promising prognostic biomarker and potential therapeutic target in breast cancer.

## Introduction

Breast cancer remains one of the most frequently diagnosed malignancies among women worldwide and continues to impose a substantial mortality burden 1. Notably, rising incidence and mortality rates are disproportionately observed in countries with lower sociodemographic index (SDI) levels 2. A comprehensive 25-year global analysis revealed increasing breast cancer mortality across most regions of the world, with the notable exception of high-income countries such as the United States and European nations, where mortality rates have steadily declined 3-5. These disparities underscore that further reductions in breast cancer-related mortality can be achieved through improved access to effective prevention strategies, early detection programs, and high-quality treatment services, as well as through identifying molecular mechanisms that drive tumor progression and therapeutic resistance 6.

At the molecular level, the ubiquitin proteasome system plays a central role in maintaining cellular proteostasis. The 26S proteasome is responsible for the regulated degradation of ubiquitinated proteins and thereby governs essential cellular processes, including DNA synthesis and repair, transcription, translation, signal transduction, metabolism, and immune regulation 7, 8. Dysregulation of these processes is closely linked to tumorigenesis, making the proteasome an attractive therapeutic target in cancer 9. Structurally, the 26S proteasome consists of a 20S catalytic core particle and one or two 19S regulatory particles 10. The 20S core is composed of two outer alpha rings, PSMA1-7, and two inner beta rings, PSMB1-7, while the 19S regulatory particle comprises ATPase, PSMC1-6, and non-ATPase subunits, PSMD1-14 11-13. Consistent with its biological importance, numerous studies have reported dysregulation of proteasome subunits as potential biomarkers or therapeutic targets in breast cancer, including members of the PSMA, PSMB, PSMC, and PSMD families 14, 15.

Proper assembly and maturation of the 20S proteasome core particle are critically dependent on proteasome assembly chaperones, also known as proteasome assembling chaperones or PACs, which are encoded by four genes: *PSMG1*, *PSMG2*, *PSMG3*, and *PSMG4* 16, 17 (Supplementary Table S1). Despite their essential role in proteasome biogenesis, the contributions of PSMG family members to breast cancer initiation and progression remain largely unexplored. Importantly, emerging evidence suggests that individual *PSMG* genes may exert distinct, context-dependent functions, acting as either oncogenes or tumor suppressors. Therefore, elucidating their expression patterns and functional roles across molecular subtypes of breast cancer is of particular significance.

Through preliminary multi-omics analyses, PSMG3 emerged as a compelling candidate for focused investigation. Compared to other PSMG family members, PSMG3 consistently exhibited the most robust oncogenic signatures across multiple large-scale datasets. Specifically, PSMG3 was significantly elevated in The Cancer Genome Atlas (TCGA) Breast Invasive Carcinoma (BRCA) cohort, displayed distinct promoter methylation patterns, and showed the significant association with unfavorable patient survival in both TCGA and Molecular Taxonomy of Breast Cancer International Consortium (METABRIC) cohorts. Moreover, elevated PSMG3 expression was preferentially observed in aggressive breast cancer subtypes, particularly triple negative breast cancer (TNBC). Co-expression network analyses further revealed that PSMG3-associated genes were enriched in pathways related to oxidative phosphorylation (OXPHOS), hypoxia responses, and immune modulation, features that were not observed to a comparable extent with other PSMG family members.

**Figure 1:**
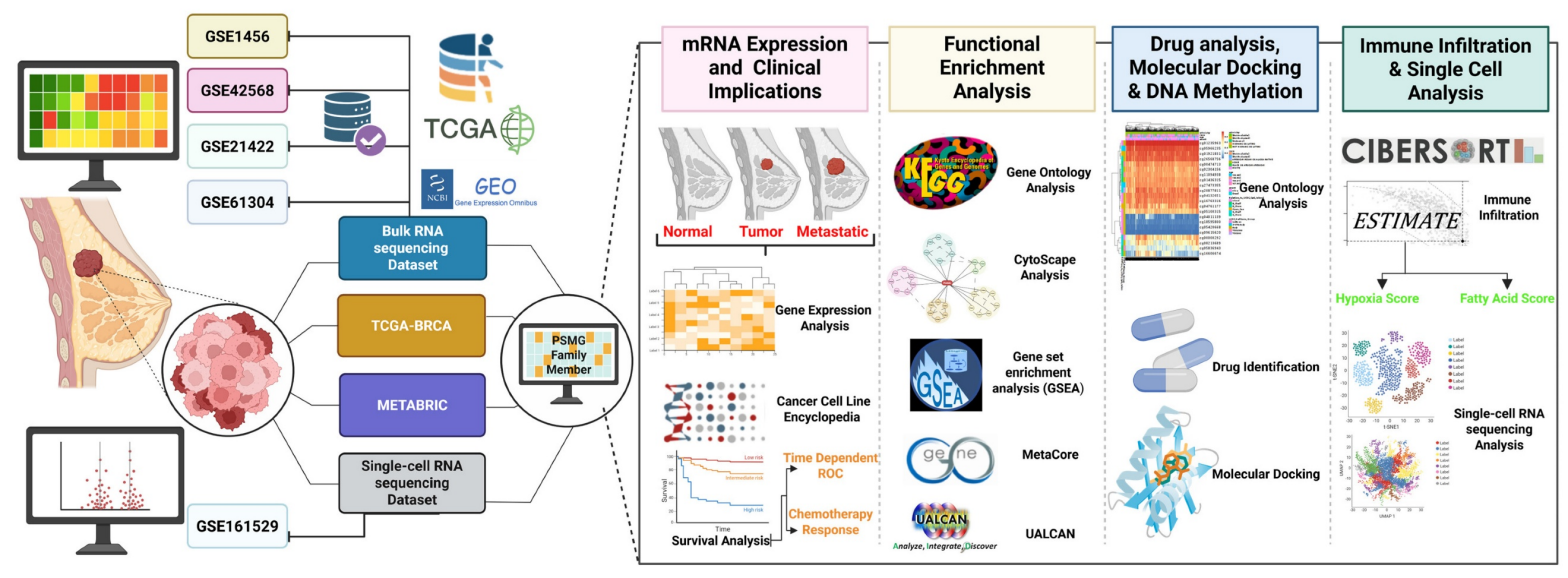
** Study workflow.** Bulk GEO datasets (GSE1456, GSE42568, GSE21422, and GSE61304) plus TCGA-BRCA/METABRIC, and scRNA-seq (GSE161529) were integrated to profile the PSMG family in breast cancer, followed by four analyses: (1) mRNA expression and clinical relevance, (2) functional enrichment (GO/KEGG/GSEA), (3) drug/docking and DNA methylation, and (4) immune infiltration and single-cell characterization.

Although previous studies reported general roles for PACs and immune metabolic regulators in cancer, no study to date has systematically investigated PSMG3 in breast cancer using integrated multi-omics approaches. In particular, comprehensive analyses combining bulk transcriptomics, tumor immune microenvironment profiling, and single cell resolution remain lacking. To address this critical knowledge gap, in the present study, we comprehensively characterized the expression landscape, clinical relevance, immune associations, and potential regulatory functions of PSMG3 in breast cancer. By integrating bulk RNA-sequencing (RNA-seq) and single-cell (sc)RNA-seq datasets, we evaluated the functional roles of *PSMG* family genes in tumor development and clinical outcomes. This integrative framework enabled a deeper understanding of the heterogeneous and subtype-specific contributions of PSMG family members to breast cancer progression 19,20 (Figure 1), and highlights PSMG3 as a promising molecular determinant with potential prognostic and therapeutic relevance.

## Materials and Methods

### Data Acquisition and Preprocessing

In this study, gene expression data were primarily obtained from TCGA-BRCA dataset 18, which was complemented by a Genotype-Tissue Expression (GTEx) dataset 19, which provides publicly available normal breast tissue samples for differentially expressed gene (DEG) analyses. Corresponding clinical data from TCGA-BRCA were collected to evaluate the association between *PSMG* gene family expressions and patient survival outcomes in breast cancer. These findings were subsequently validated using independent external cohorts, including Molecular Taxonomy of Breast Cancer International Consortium (METABRIC), a publicly accessible breast cancer dataset from the European Genome-Phenome Archive 20, as well as Gene Expression Omnibus (GEO) microarray datasets GSE1456 21, GSE42568 22, GSE21422 23, and GSE61304 24, which were used to confirm the DEG results observed in TCGA-BRCA cohort. In addition, the investigation was extended to single-cell resolution by incorporating the GSE161529 Single-cell RNA sequencing (scRNA-seq) dataset 25 to examine the specific contribution of *PSMG* genes to tumor progression at the cellular level. Detailed characteristics of all included datasets are summarized in Table 1.

To ensure the reproducibility and robustness of our findings across diverse platforms, a rigorous data processing and harmonization strategy was implemented. For RNA-seq datasets, including TCGA-BRCA, gene expression values were normalized using the transcripts per million (TPM) method and then subsequently log2-transformed to stabilize the variance 26-28. For microarray datasets (GSE42568, GSE21422, GSE61304, and GSE1456), raw intensity data were processed using the Robust Multi-array Average (RMA) algorithm, followed by quantile normalization 29-31. To minimize batch effects, our primary analytical strategy emphasized independent cross-dataset validation rather than direct data pooling. Associations were established in the training cohort (TCGA-BRCA) and independently validated in each external cohort. For analyses requiring a combined test dataset, Z-score normalization was applied within each dataset prior to integration to mitigate platform-specific baseline differences while preserving relative expression patterns. The detailed characteristics of all incorporated datasets are summarized in Table 1.

### Expression Patterns of PSMGs in Breast Cancer

Normalized RNA-seq expression data for *PSMG* family genes were downloaded from TCGA-BRCA dataset. This cohort includes normal breast tissue samples, primary breast tumor samples, and seven metastatic breast tumor samples. Expression levels of *PSMG* genes were systematically compared among normal, primary tumors, and metastatic tissues to characterize their expression patterns during breast cancer progression.

### Cancer Cell Line Encyclopedia (CCLE) Analysis

To complement the analysis of *PSMG* messenger (m)RNA expression data from the TNMplot database, we further evaluated their expressions across breast cancer cell lines using the CCLE database (https://portals.broadinstitute.org/ccle) 32. The CCLE is a comprehensive web-based resource that provides pharmacologic and genomic characterizations of diverse human cancer models. RNA-seq-aligned read data were extracted from 51 breast cancer cell lines using default parameters, as previously described 33, 34. Breast cancer cell lines were categorized into molecular subtypes, including luminal A, luminal B, human epidermal growth factor receptor 2 (HER2)-enriched, TNBC, and other subtypes.

### Survival Analysis (Kaplan-Meier (KM) Plot)

KM survival analyses were performed to evaluate the associations between *PSMG* mRNA expression levels and patient survival outcomes. Three independent datasets were used. First, the Kaplan-Meier Plotter database (https://kmplot.com/) was employed, which integrates gene expression and survival data from breast cancer patients derived from GEO microarray platforms (Affymetrix HGU133A and HGU133 Plus 2.0) 35. Survival endpoints included distant metastasis-free survival (DMFS) and relapse-free survival (RFS), with patients stratified into high- and low-expression groups based on median PSMG expression. Hazard ratios (HRs) with 95% confidence intervals (CIs) and log-rank *p* values were calculated. Second, survival associations were validated using the METABRIC cohort. Corresponding *PSMG* mRNA expression and clinical data were obtained from the cBioPortal database 36. Survival analyses for validation datasets were conducted using the R package *survival* 37-41. Finally, the prognostic significance of PSMG3 was further validated in the GSE1456 dataset, which included 159 breast cancer patients.

### DNA Methylation, Protein Expression, and UALCAN Validation

Genome-wide DNA methylation data and corresponding survival information for breast invasive carcinoma were obtained using the MethSurv platform (https://biit.cs.ut.ee/methsurv/) 42. These data were derived from TCGA and generated using the Illumina Infinium HumanMethylation450 BeadChip (HM450K), which covers cytosine guanine (CpG) sites across the *PSMG3* gene, including transcription start sites, CpG islands, and adjacent regulatory regions. To validate the relationship between PSMG3 DNA methylation and protein expression, PSMG3 protein levels in normal breast tissues and breast cancer tissues were examined using the Human Protein Atlas (HPA) database (https://www.proteinatlas.org/) 43. Immunohistochemical (IHC) data from formalin-fixed, paraffin-embedded tissue microarrays were analyzed using HPA-validated antibodies. Protein expression was semi-quantitatively assessed by certified pathologists based on the staining intensity (negative, low, medium, or high) and the proportion of positive cells 44-46. Additionally, the UALCAN portal (http://ualcan.path.uab.edu/) 47 was used to validate promoter DNA methylation differences between normal and primary breast tumor tissues, providing independent confirmation of methylation alterations.

### Functional Enrichment Analysis

To investigate the functional relevance of PSMG3, functional enrichment analyses were conducted using the clusterProfiler R package 48. TCGA-BRCA samples were divided into high- and low-expression groups based on the median PSMG3 expression level 49-51. DEGs between the two groups were analyzed for gene ontology (GO) biological processes, molecular functions, and cellular components. A gene set enrichment analysis (GSEA) was performed using Hallmark gene sets to identify pathway-level alterations 52-56. To further explore PSMG3-associated biological networks, co-expressed genes were identified in three datasets: TCGA-BRCA, METABRIC, and a TNBC cohort. Co-expression gene lists were obtained from cBioPortal using a correlation threshold of > 0.25 and a *p* value of < 0.05. A three-way overlap analysis was performed, yielding 17,238 commonly co-expressed genes across all cohorts. This core gene set was subsequently analyzed using MetaCore software 57-59 to identify significantly enriched signaling pathways and biological processes based on curated pathway maps.

### Gene Set Signature Analysis and Immune Deconvolution

Immune deconvolution analyses were conducted to assess the tumor immune cell composition and its relationship with PSMG expression. CIBERSORT 60 was used to estimate the relative proportions of immune cell types from bulk RNA-seq data. ESTIMATE 61 was applied to calculate immune scores, stromal scores, and tumor purity, which served as independent validation of immune infiltration results. A gene set variation analysis (GSVA) 62 was employed to evaluate pathway-level activity across samples. All immune and pathway analyses were implemented using the IOBR R package 63, which integrates these computational frameworks. These analyses elucidate the immune, hypoxic, and tumor microenvironmental (TME) contexts associated with *PSMG* gene expressions.

### Transcriptional Heterogeneity in Breast Cancer Using a Single-Cell Analysis

To investigate PSMG3 expression within the context of breast cancer heterogeneity, scRNA-seq data from GSE161529 were analyzed. This dataset includes six normal breast tissue samples from premenopausal and postmenopausal donors and four TNBC samples. Data processing and analysis were conducted using the Seurat R package 64. Cells were filtered to remove those with fewer than 500 detected genes or more than 20% mitochondrial gene content. Gene expression values were normalized using the LogNormalize method. A principal component analysis (PCA) was performed for dimensionality reduction, and the top 20 principal components were selected based on an elbow plot after identifying 2000 highly variable genes using the vst method 65-67. Cell types were annotated using the SingleR package 68 and validated against established marker genes from prior studies 69.

### Drug Sensitivity Analysis and Molecular Docking

Drug sensitivity associated with PSMG3 expression was predicted using data from the Genomics of Drug Sensitivity in Cancer (GDSC) and Cancer Therapeutics Response Portal (CTRP) databases 70. These resources integrate genomic profiles from over 33 cancer types and drug response data for more than 750 compounds 71-73. PSMG3-related drug sensitivity profiles were extracted for a downstream analysis. To evaluate potential molecular interactions between PSMG3 and candidate therapeutic compounds, molecular docking simulations were performed. The crystal structure of PSMG3 (PDB ID: 2Z5E) was obtained from the RCSB Protein Data Bank. Potential ligand-binding pockets were predicted using PrankWeb 74. Docking simulations were conducted using AutoDock Vina, which predicts optimal binding conformations and binding affinities based on an efficient scoring algorithm suitable for high-throughput screening 75.

### Statistical Analysis

All statistical analyses were performed using R software vers. 4.1.3. Differences between groups were evaluated using the Wilcoxon rank-sum test. Multiple test corrections were applied using the false discovery rate (FDR) method, and an adjusted *p* value of < 0.05 was considered statistically significant 76-78. A detailed schematic of the analytical workflow and computational procedures is provided in Figure 1 and Supplementary Figure S1.

## Results

### Differential Expression of PSMG Family Genes in Breast Cancer

**Figure 2:**
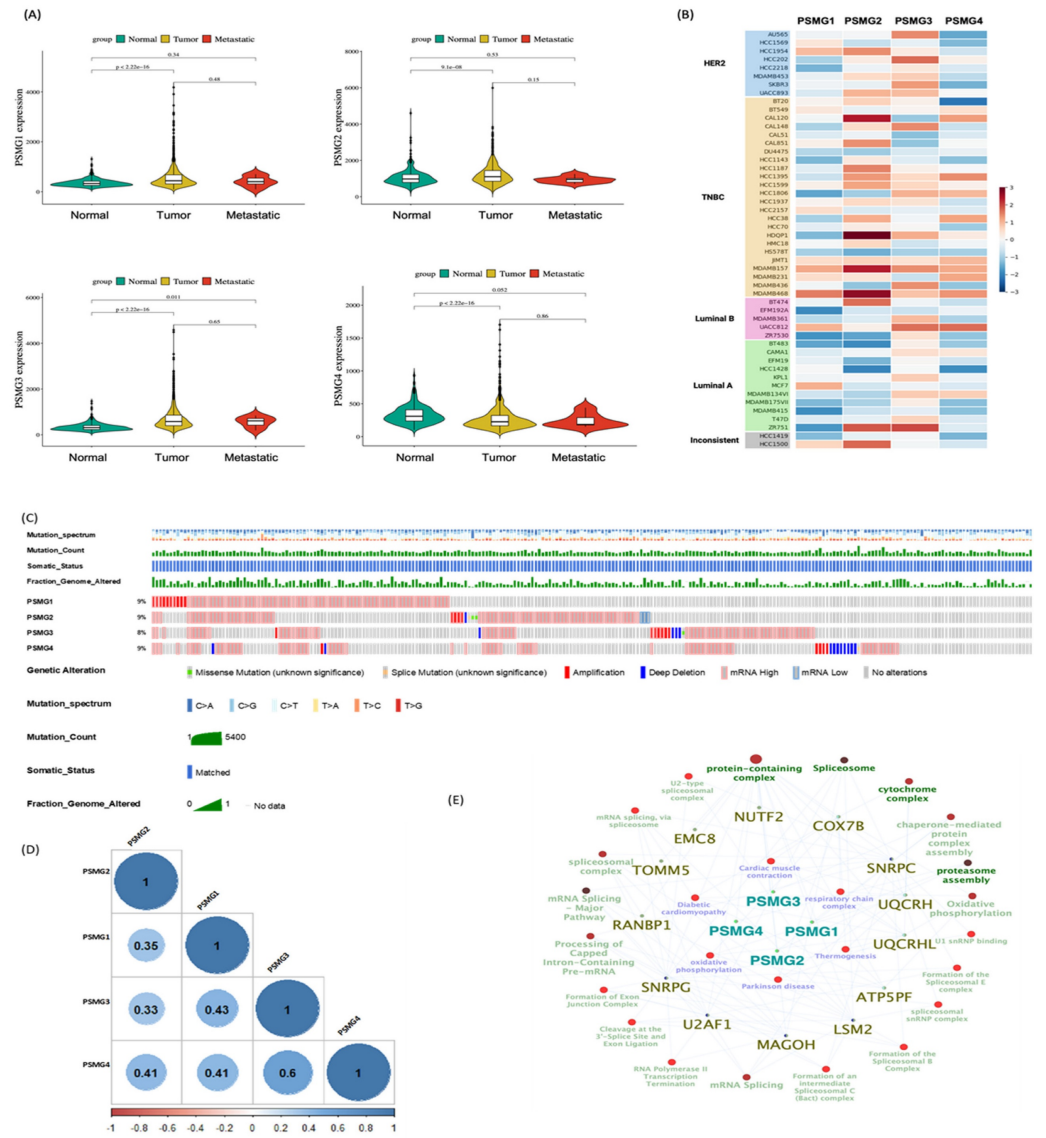
** Characterization of *PSMG* family expressions and genomic features in breast cancer.** (A) Violin plots showing normalized mRNA expressions of *PSMG1*-*PSMG4* in TCGA-BRCA cohort across normal, primary tumor, and metastatic samples; *p* values are shown, and significance was defined as *p* < 0.05. (B) Heatmap of PSMG1-PSMG4 expressions across breast cancer cell lines (grouped by subtype, such as HER2, TNBC, luminal A/B). (C) Oncoprint-style summary of PSMG genomic alterations in TCGA-BRCA. Red indicates copy-number amplification and blue indicates deep deletion. (D) Pairwise correlation matrix of PSMG1-PSMG4 expressions in breast cancer (visualized with the corrplot R package); non-significant correlations are marked with an “×” (*p* > 0.01). (E) Pathway-gene network based on the METABRIC dataset, generated in Cytoscape using ClueGO. Pathways meeting the enrichment threshold (*p* ≤ 0.05) are displayed; enrichment was tested using a two-sided hypergeometric test with the Bonferroni step-down correction.

Given the limited evidence regarding the involvement of the *PSMG* gene family in breast cancer, we first conducted a pan-cancer expression analysis with a specific focus on breast malignancies. Analysis of TCGA-BRCA cohort revealed that *PSMG1* and *PSMG3* were significantly elevated in breast cancer tissues, whereas *PSMG2* and *PSMG4* were downregulated relative to normal breast tissues (Figure 2A). To validate these findings, we examined independent breast cancer microarray datasets (GSE21422, GSE42568, and GSE61304). Consistent with TCGA-BRCA results, PSMG3 exhibited a reproducible trend of elevated expression in tumor samples across all three datasets, although effect sizes varied between cohorts (Supplementary Figure S2). In contrast, PSMG2 and PSMG4 consistently displayed reduced or unchanged expression, supporting their downregulation. PSMG1 showed modest and dataset-dependent changes, suggesting potential context-specific regulation. Volcano plots and box plots collectively demonstrated the robustness of PSMG3's dysregulation across multiple independent cohorts, reinforcing its relevance in breast cancer biology. We next assessed PSMG expressions across breast cancer cell lines using CCLE data. PSMG2 showed relatively high expression in TNBC cell lines. Notably, PSMG3 was highly expressed in both non-TNBC cell lines (AU565 and HCC202) and several TNBC cell lines (CAL148, HCC1806, MDA-MB-157, and MDA-MB-436), whereas PSMG4 displayed uniformly low expression across most cell lines (Figure 2B). To further investigate clinical relevance, a stratified expression analysis using the UALCAN platform demonstrated that PSMG3 expression was significantly elevated across all major molecular subtypes of breast cancer, with the highest expression observed in TNBC (Supplementary Figure S3). PSMG3 expression was also significantly higher in TP53-mutant tumors than in TP53 wild-type tumors, suggesting an association with genomic instability. A stage-wise analysis revealed a progressive increase in PSMG3 expression from early to advanced disease stages, and elevated expression was consistently observed across menopausal status and nodal metastasis subgroups. Collectively, these findings suggested that PSMG3 upregulation is broadly associated with breast cancer progression and aggressive clinicopathological features.

### Genetic Alterations and Co-Expression Analysis of PSMG Family Genes

To characterize the genetic landscape of the *PSMG* family, we analyzed *PSMG* gene mutations in breast cancer patients using TCGA Pan-Cancer Atlas dataset, revealing mutation frequencies ranging 8%-9% (Figure 2C). We further examined relationships between *PSMG* gene expressions and other genes using mRNA expression data from TCGA-BRCA cohort, specifically focusing on breast cancer samples (Figure 2D). Co-expression analyses were then performed to identify genes significantly correlated with *PSMG* family members, yielding a final set of 525 genes for subsequent a gene ontology enrichment analysis (Figure 2E). This integrative approach enabled the identification of functional associations and biological processes linked to *PSMG* family genes, thereby providing insights into their potential roles in breast cancer pathogenesis.

### Survival Analysis and Protein Expressions of PSMG Family Genes

**Figure 3:**
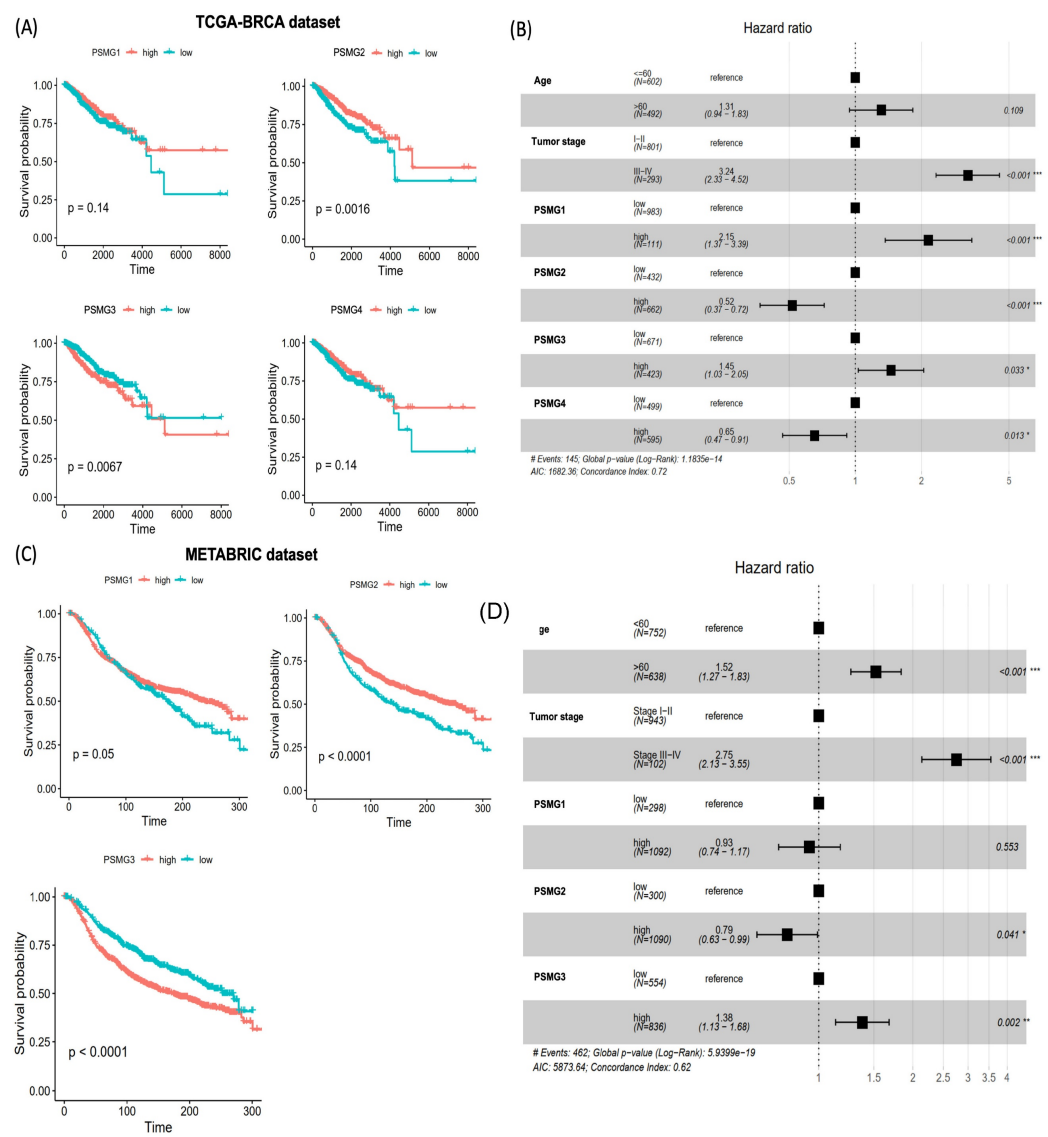
** Prognostic value of *PSMG* family gene expressions in breast cancer.** (A) Kaplan-Meier survival curves for *PSMG1*-*PSMG4* in TCGA-BRCA cohort, comparing high versus low expression groups; *p* values were calculated by the log-rank test. (B) Forest plot of multivariate Cox proportional hazards models in TCGA-BRCA including age, tumor stage, and PSMG1-PSMG4 expressions. Squares denote hazard ratios (HRs) and horizontal lines indicate 95% confidence intervals (CIs). (C) Kaplan-Meier survival validation of PSMG1-PSMG3 in the METABRIC cohort (*n* = 1390), stratified by high versus low expression. (D) Multivariate Cox regression in METABRIC assessing the independent prognostic effects of *PSMG* genes after adjusting for clinical covariates; HRs with 95% CIs are shown.

To evaluate associations between *PSMG* mRNA expressions and patient survival in breast cancer, KM survival analyses were performed using TCGA-BRCA dataset. Patients were stratified into high- and low-expression groups based on optimal cutoff points determined using the surv_cutpoint function from the survminer R package. A univariate analysis revealed that elevated PSMG2 expression was associated with a favorable prognosis (*p* = 0.0016), whereas high PSMG3 expression was significantly correlated with poorer overall survival (OS) (*p* = 0.0067). In contrast, no significant associations with survival were observed for PSMG1 or PSMG4 in the univariate analysis (Figure 3A). To further assess the independent prognostic value of individual PSMG family members, multivariate Cox proportional hazards regression analyses were conducted after adjusting for relevant clinical covariates. High expression of PSMG1 (*p* < 0.001) and PSMG3 (*p* = 0.033) emerged as independent predictors of poor survival, whereas PSMG2 (*p* < 0.001) and PSMG4 (*p* = 0.013) were identified as independent protective factors (Figure 3B). These findings were further validated using the METABRIC cohort, which consistently demonstrated that high PSMG3 expression was associated with poorer survival outcomes, both in the univariate analysis (Figure 3C) and in multivariate models incorporating additional clinical variables (Figure 3D). Collectively, these results highlighted the clinical relevance of the PSMG family as prognostic biomarkers in breast cancer, with PSMG3 emerging as a robust and independent predictor of patient outcomes.

### Transcriptomic and Epigenetic Characterization of PSMG3

**Figure 4:**
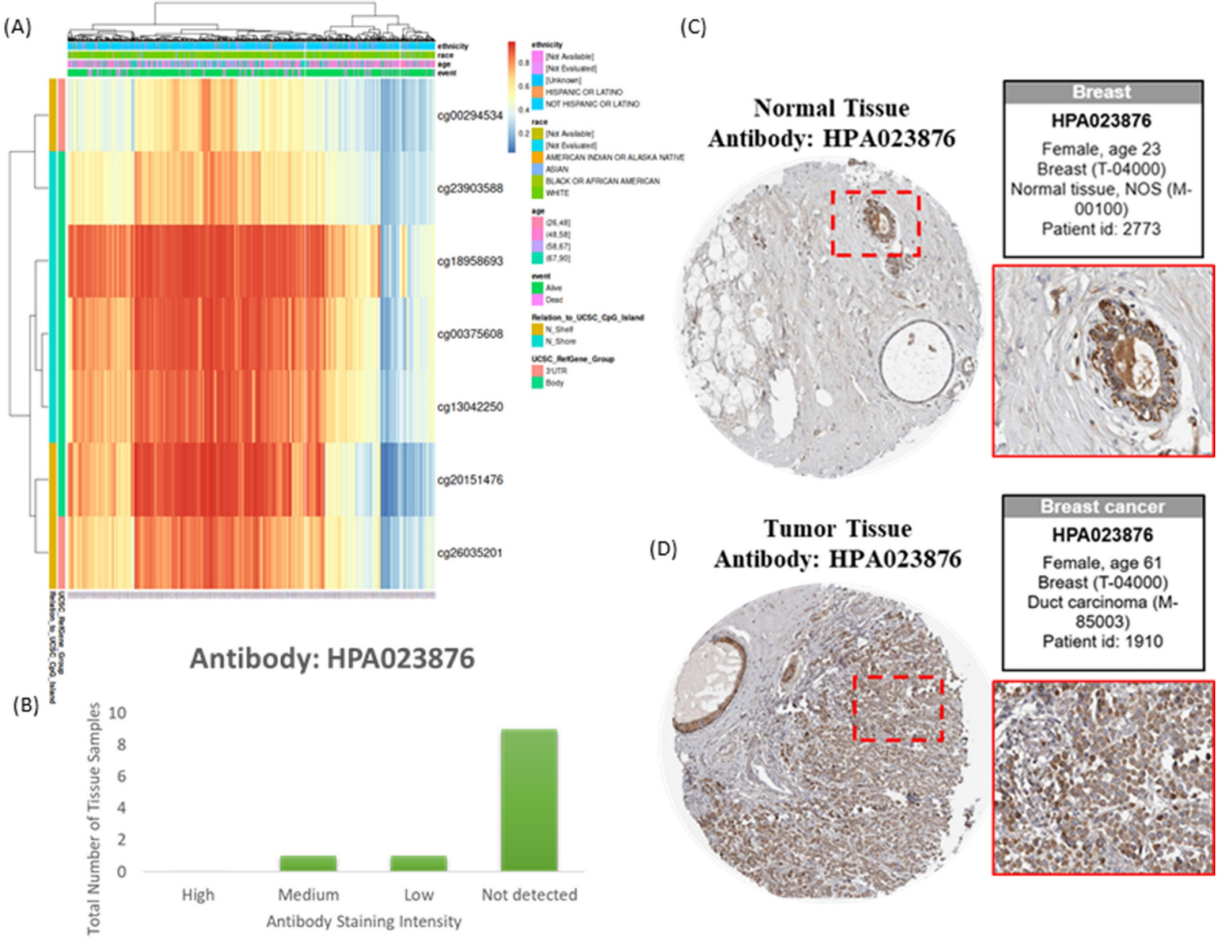
** Epigenetic and protein-level characterization of PSMG3 in breast cancer.** (A) Heatmap showing methylation levels of PSMG3-associated CpG sites across samples, with corresponding clinical/demographic annotations. (B) Distribution of PSMG3 immunohistochemical (IHC) staining intensities across breast tissue samples from the Human Protein Atlas (antibody HPA023876). (C, D) Representative IHC images (HPA023876) illustrating low/weak PSMG3 staining in normal breast tissues (C) and increased PSMG3 immunoreactivity in breast cancer tissues (D). Insets show magnified views of the boxed regions.

Integration of transcriptomic and DNA methylation data revealed that several CpG sites, including cg00294534 and cg23903588, were significantly correlated with PSMG3 expression (Figure 4A). These epigenetic signatures suggested that aberrant DNA methylation contributes to PSMG3 dysregulation. Consistent prioritization across TCGA, METABRIC, and GEO cohorts identified PSMG3 as the only PSMG family member consistently elevated and independently associated with poor survival. An IHC analysis further supported these findings, showing minimal PSMG3 protein expression in normal breast tissues (Figure 4C) but higher staining in malignant cells of invasive ductal carcinoma (Figure 4D). The distribution of the staining intensity is summarized in Figure 4B. Functional integration of these data linked PSMG3 expressions with ErbB2 signaling and heat shock protein 90 (Hsp90) dependency pathways, supporting its role as an active regulator rather than a passive marker of tumor aggressiveness.

### Diagnostic Performance of PSMG Family Genes

**Figure 5:**
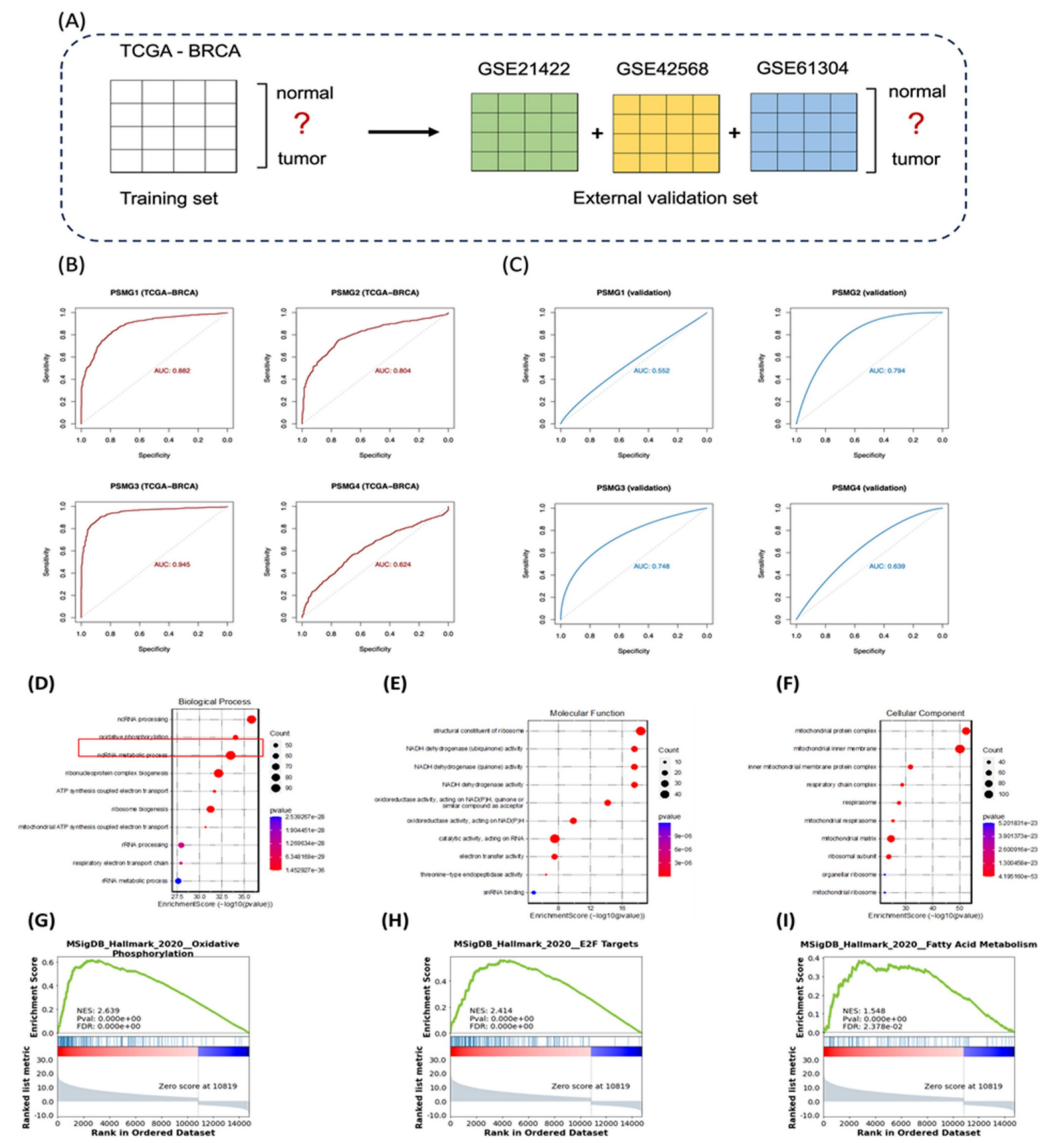
** Diagnostic performance and functional enrichment analysis of *PSMG* family genes in breast cancer.** (A) Study design schematic showing TCGA-BRCA as the training cohort (normal vs. tumor) and GSE21422, GSE42568, and GSE61304 as external validation cohorts. (B) ROC curves assessing the diagnostic accuracy of PSMG1-PSMG4 in TCGA-BRCA; AUC values indicate performance. (C) ROC curves validating diagnostic performance in the combined external GEO cohort. (D-F) GO enrichment of *PSMG3*-associated DEGs, including (D) biological process, (E) molecular function, and (F) cellular component categories. (G-I) GSEA plots comparing high- vs low-PSMG3 groups, highlighting enriched Hallmark pathways including (G) oxidative phosphorylation, (H) E2F targets, and (I) fatty acid metabolism.

Logistic regression models were constructed using TCGA-BRCA as the training cohort, with external validation performed with the GSE42568, GSE21422, and GSE61304 datasets (Figure 5A). Among all PSMG family members, PSMG3 demonstrated the highest diagnostic accuracy in the training set (area under the curve (AUC) = 0.945) and maintained robust performance in external validation (AUC = 0.748). These results were further confirmed by five-fold cross-validation (Figure 5B, C), highlighting PSMG3 as the most promising diagnostic candidate.

### Functional Enrichment Analysis of PSMG3

TCGA-BRCA patients were stratified into high- and low-expression groups based on the median PSMG3 expression level. DEGs between the two groups were identified and subsequently subjected to a GO enrichment analysis using the clusterProfiler R package (Figure 5D-F). Notably, the GO analysis revealed OXPHOS, a key mitochondria-related cellular pathway, and electron transfer activity as the most significantly enriched terms across the biological process, cellular component, and molecular function categories. To further validate these biological associations, a GSEA was performed on the pre-ranked DEG list using the Hallmark gene set database. This analysis identified several gene signatures significantly enriched in the high-PSMG3 expression group compared to the low-expression group. Consistent with the GO results, OXPHOS emerged as a prominently enriched pathway, along with additional signatures such as E2F targets and fatty acid (FA) metabolism (Figure 5G-I).

### Co-Expression Network and Pathway Validation

**Figure 6:**
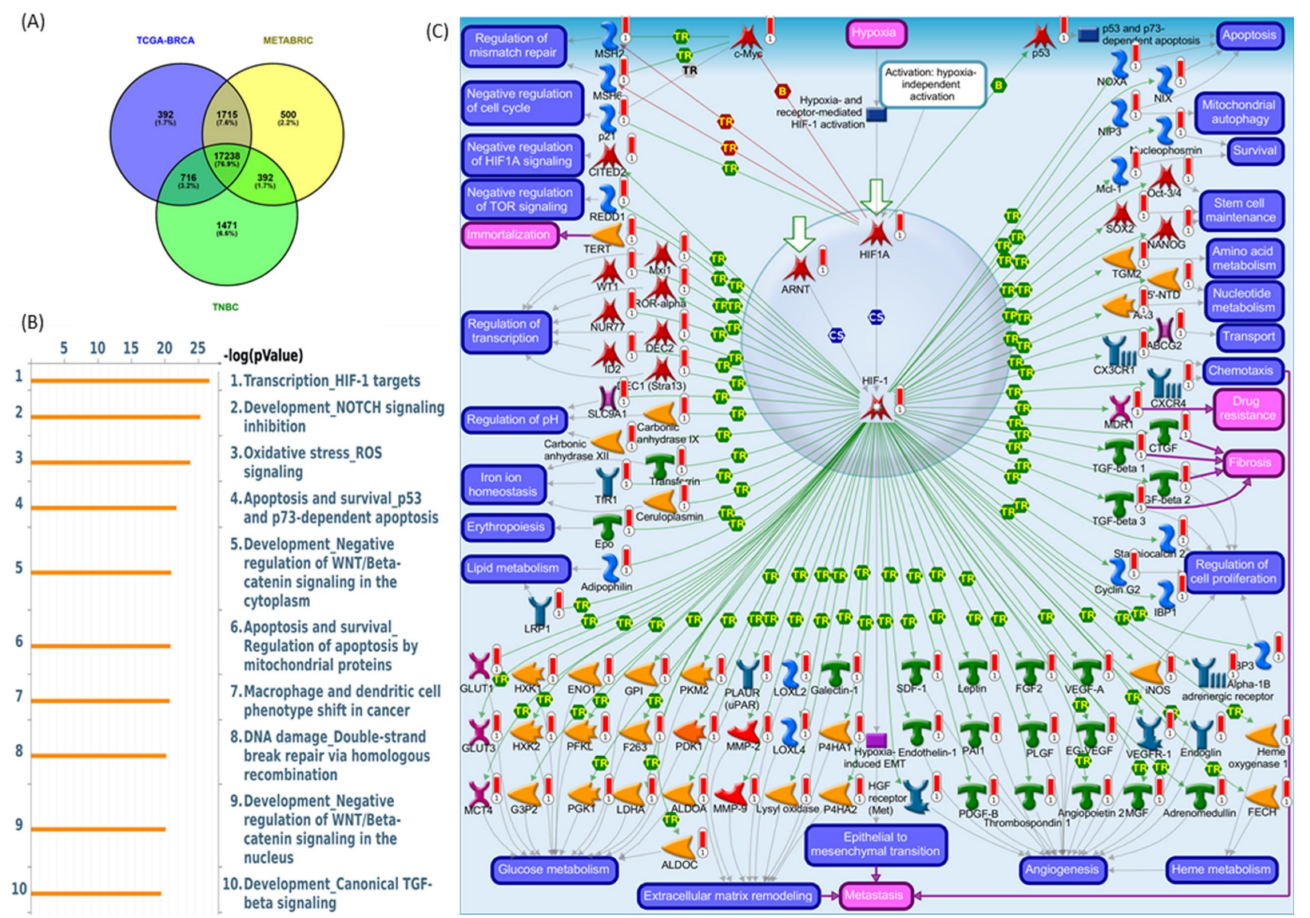
**Correlation and pathway analysis of overlapping *PSMG3*-associated genes.** (A) Venn diagram showing the overlap of differentially expressed genes (DEGs) among TCGA-BRCA, METABRIC, and TNBC, identifying a shared core gene set. (B) MetaCore pathway enrichment of shared genes. The top 10 significantly enriched pathways are ranked by -log(*p*), with HIF-1 target transcription as the leading signal. (C) MetaCore network map of HIF-1 signaling, highlighting interconnected genes and regulatory modules involved in hypoxia responses, apoptosis/survival, metabolic rewiring, and angiogenesis across breast cancer subsets.

To delineate the core transcriptional programs driven by the PSMG3 PAC across heterogeneous breast cancer contexts, we performed a stringent three-way co-expression analysis. Genes positively correlated with PSMG3 expression (Pearson correlation coefficient > 0.25, *p* < 0.05) were independently identified in three clinically distinct and well-characterized breast cancer cohorts: TCGA-BRCA, METABRIC, and a TNBC dataset. Intersection of these gene sets yielded a convergent core signature comprising 17,238 overlapping genes, representing a transcriptional program consistently associated with PSMG3 across multiple breast cancer subtypes (Figure 6A). Identification of this shared gene set minimized dataset-specific noise and cohort-related variability, thereby highlighting pathways of broad biological relevance. A subsequent pathway enrichment analysis using MetaCore software prioritized key functional signaling networks within this core gene set, with the “Transcription, HIF-1 targets” pathway emerging as the most significantly enriched (Figure 6B). The corresponding canonical pathway map illustrates critical regulatory nodes and downstream effector genes governed by the hypoxia-inducible factor-1A (HIF1A)/ aryl hydrocarbon receptor nuclear translocator (ARNT) transcriptional complex (Figure 6C).

Biologically, these findings suggest that PSMG3-driven oncogenic processes are closely linked to hypoxia-responsive signaling. Under hypoxic conditions, HIF1A becomes stabilized, heterodimerizes with ARNT, and is translocated to the nucleus to activate transcriptional programs that promote cellular adaptation to the TME. These programs regulate key malignant processes, including metabolic reprogramming, resistance to apoptosis, enhanced cell survival, and angiogenesis. A complete list of the top enriched MetaCore pathway maps (ranks 2-10) is provided in Supplementary Figures S4-S12. This inferred biological framework was further supported by functional and clinical validation. A GSEA of the PSMG3-associated transcriptional signature demonstrated significant enrichment of Hallmark pathways, particularly OXPHOS and MSigDB Hallmark E2F Targets. Enrichment of OXPHOS suggests that despite the association with hypoxia signaling, the PSMG3 signature is also linked to elevated metabolic demands required for sustained proliferation. Concurrent enrichment of E2F target genes directly implicates PSMG3 in deregulated cell-cycle progression, a hallmark of aggressive tumor behavior. In addition, a complete list of significantly repressed co-expressed genes is provided in Supplementary Table S2.

### Immune Deconvolution and TME Analysis

**Figure 7:**
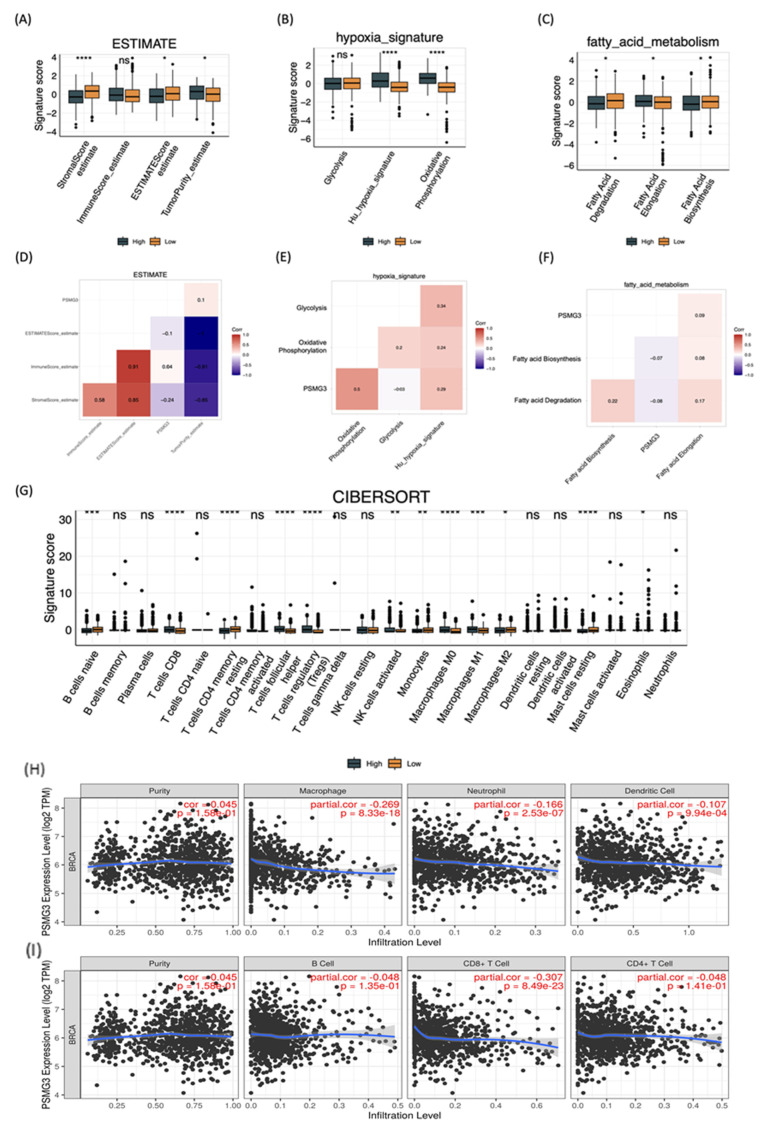
** Tumor microenvironment remodeling, metabolic reprogramming, and immune infiltration associated with PSMG3 expression in breast cancer.** (A-C) Boxplots comparing pathway/signature scores between PSMG3-high and PSMG3-low groups: (A) ESTIMATE-derived stromal score, immune score, ESTIMATE score, and tumor purity; (B) hypoxia-related programs (glycolysis, hypoxia signature, and oxidative phosphorylation); and (C) fatty-acid metabolism modules (fatty-acid degradation, elongation, and biosynthesis). (D-F) Correlation heatmaps showing relationships among PSMG3 expression, microenvironment features, and metabolic signatures: (D) ESTIMATE stromal/immune/purity metrics; (E) hypoxia-associated metabolic programs; and (F) fatty-acid metabolism pathways (red, positive; blue, negative correlations). (G) CIBERSORT comparison of 22 tumor-infiltrating immune cell fractions between PSMG3-high and PSMG3-low tumors; significance is denoted as *p* < 0.05, *p* < 0.01, *p* < 0.001, *p* < 0.0001; ns, not significant. (H, I) TIMER scatterplots of PSMG3 expression versus immune infiltration levels in TCGA-BRCA, reporting purity-adjusted partial correlations (r) and *p* values. Negative associations were observed with multiple immune populations (such as macrophages, neutrophils, dendritic cells, B cells, and CD8⁺ T cells). Blue lines indicate fitted linear regression.

We first investigated the impact of PSMG3 expression on the TME and stromal composition using the ESTIMATE algorithm. High PSMG3 expression was significantly associated with lower stromal and ESTIMATE scores (Figure 7A, D), indicating a reduced non-tumor stromal content. Consistently, tumors in the high-PSMG3 group exhibited significantly higher tumor purity scores (Figure 7A, D), suggesting an increased proportion of malignant cells within the tumor mass. These findings position PSMG3 as a marker potentially linked to enhanced tumor cell dominance in breast cancer. To explore the relationship between PSMG3 expression and metabolic reprogramming, pathway activity scores were compared between high- and low-PSMG3 expression groups. Consistent with the MetaCore analysis identifying “Transcription, HIF-1 targets” as a top pathway, high PSMG3 expression was associated with increased activities of key metabolic programs, including glycolysis, hypoxia signaling, and OXPHOS (Figure 7B, E).

The concurrent enrichment of both glycolytic/hypoxic pathways and OXPHOS suggests that PSMG3 may support a metabolically flexible and aggressive tumor phenotype, enabling cancer cells to efficiently utilize both anaerobic and aerobic energy pathways. In addition, the high-PSMG3 cohort displayed significantly elevated scores for FA degradation and FA elongation relative to the low-expression group (Figure 7C, F), indicating extensive lipid metabolic reprogramming. This metabolic shift likely provides the energy supply and biosynthetic precursors necessary to sustain the high proliferative capacity inferred from prior GSEA results. Finally, immune cell deconvolution using the CIBERSORT algorithm (Figure 7G) revealed that tumors with high PSMG3 expression were enriched in multiple immune cell populations, including cluster of differentiation 8-positive (CD8⁺) cytotoxic T cells, follicular helper T cells, regulatory T cells (Tregs), natural killer (NK) cells, and both M0 and M1 macrophages. The simultaneous enrichment of effector immune cells (CD8⁺ T cells) and immunosuppressive populations (such as Tregs and M0 macrophages) suggests that high PSMG3 expression is associated with an “inflamed” yet tightly regulated immune microenvironment, characterized by active antitumor immunity counterbalanced by immunosuppressive mechanisms.

### External Validation and Single-Cell Analysis

**Figure 8:**
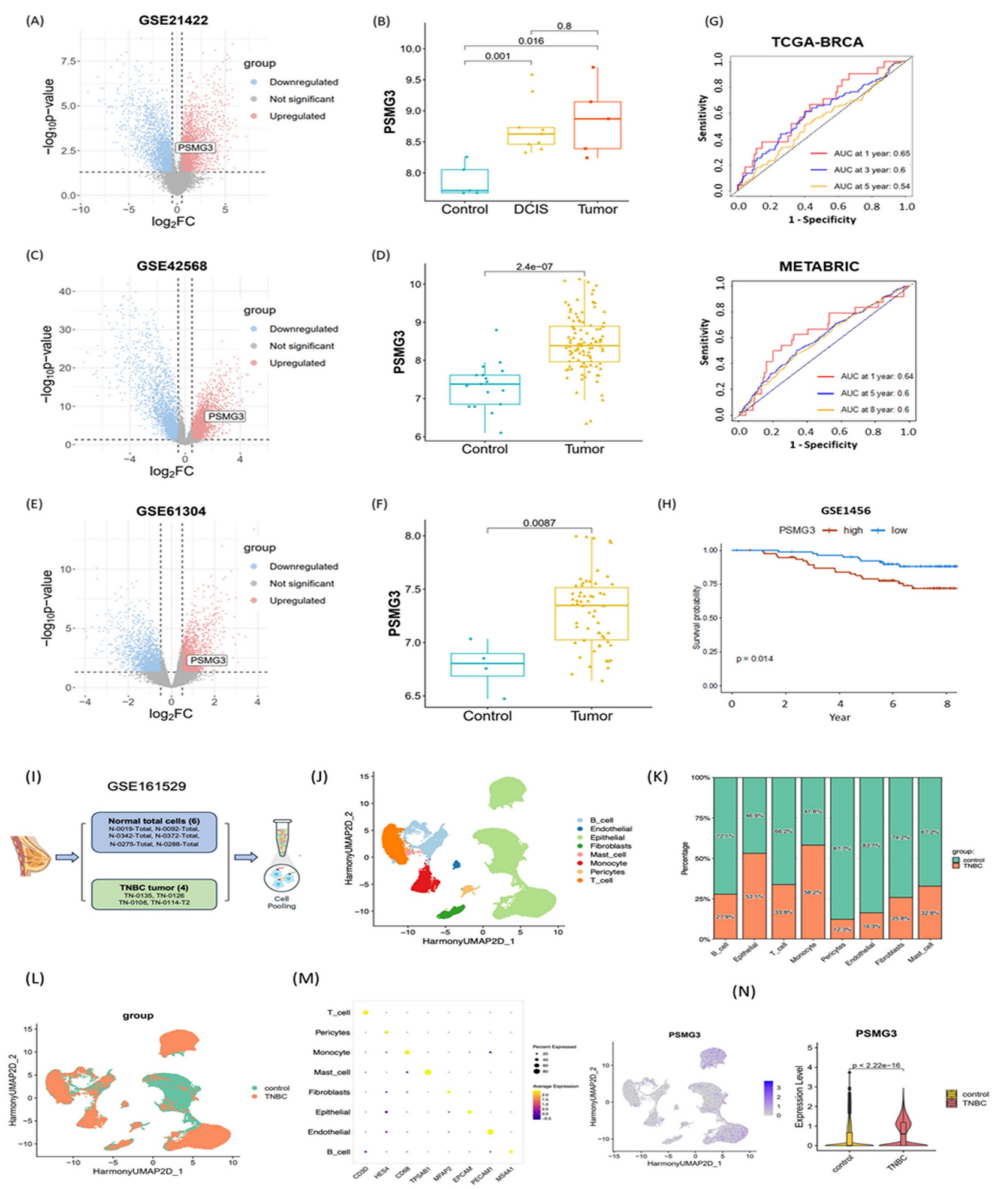
** External validation of PSMG3 expression and prognostic relevance using bulk and single-cell datasets.** (A-F) PSMG3 differential expression in independent GEO cohorts: GSE21422 (A, B), GSE42568 (C, D), and GSE61304 (E, F). Left panels show volcano plots; right panels show group comparisons (box/violin plots), consistently indicating PSMG3 upregulation in tumors versus controls (two-sided tests; *p* values shown). (G) Time-dependent ROC curves assessing the prognostic performance of PSMG3 in TCGA-BRCA (1-, 3-, and 5-year) and METABRIC (1-, 5-, and 8-year) cohorts. (H) Kaplan-Meier analysis in GSE1456 confirming poorer overall survival in the PSMG3-high group (*p* = 0.014, log-rank test). (I) Overview of the scRNA-seq analysis pipeline for GSE161529, comparing TNBC and normal breast samples. (J) UMAP embedding of integrated single cells, annotated into major cell types. (K) Stacked bar plot showing the relative abundance of each cell type in control versus TNBC samples. (L) UMAP colored by sample group (control vs. TNBC). (M) Dot plot of canonical marker genes used to support cell-type annotations. (N) Single-cell PSMG3 expression: feature plot showing cluster-level distribution (left) and violin plot showing higher expression in TNBC epithelial cells than controls (two-sided test; *p* value shown).

**Figure 9:**
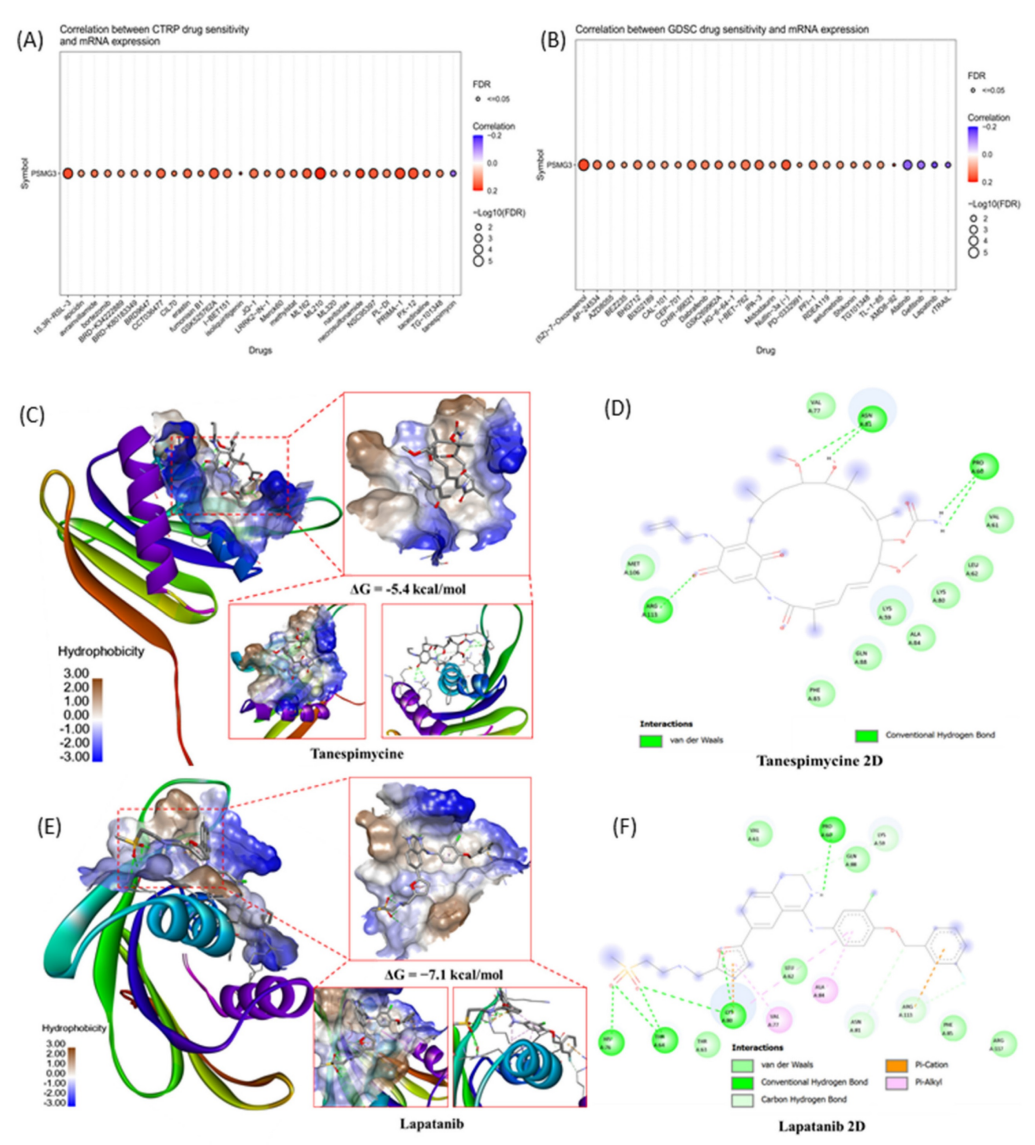
** Drug sensitivity prediction and molecular docking validation for PSMG3-targeting candidates.** (A, B) Bubble plots showing correlations between *PSMG3* mRNA expression and drug sensitivity (IC_50_) across cancer cell lines using the CTRP (A) and GDSC (B) datasets. Dot color indicates the correlation direction/strength, and dot size reflects statistical significance (-log10 FDR); FDR < 0.05 was considered significant. Drugs with negative correlations suggest increased sensitivity in PSMG3-high cells (e.g., lapatinib and tanespimycin). (C-F) Molecular docking of candidate compounds with the PSMG3 protein. C and E show 3D docking poses and binding-pocket surfaces for tanespimycin and lapatinib, respectively. D and F show corresponding 2D interaction maps highlighting key ligand-residue contacts, including hydrogen bonds and hydrophobic/π interactions, supporting the predicted pharmacological associations.

We first investigated the relationship between PSMG3 expression and tumor immune infiltration in breast cancer. Our analysis revealed that higher PSMG3 levels were negatively correlated with CD8+ T cells, macrophages, neutrophils, and dendritic cell infiltration (Figure 7H, I), suggesting that PSMG3 may be involved in modulating the tumor immune microenvironment. Next, to ensure the robustness of our observations, we validated PSMG3 expression in three independent external breast cancer datasets (GSE42568, GSE21422, and GSE61304). Consistently with our training data, PSMG3 levels were significantly higher in tumor tissues compared to normal samples across all cohorts (Figure 8A-F). In contrast, other PSMG family members showed no significant differences. We further elevated the prognostic value of PSMG3 in GSE1456 (Supplementary Figure S13). High PSMG3 expression was significantly associated with poor survival outcomes (*p* = 0.014; Figure 9H), consistent with our findings in TCGA-BRCA and METABRIC datasets. The predictive capability of PSMG3 was moderate in TCGA-BRCA (AUC: 0.65 at 1 year, 0.6 at 3 years) and comparable in the METABRIC dataset (AUC: 0.64 at 1 year, 0.6 at 5 and 8 years; Figure 8G). Finally, given the correlation observed between PSMG3 and TNBC subtypes, we sought to comprehensively characterize PSMG3 expression at a single-cell level using the GSE161529 dataset. After rigorous quality filtering, we analyzed 29,043 normal cells and 20,550 TNBC cells, identifying eight distinct cell clusters validated by canonical markers (Figure 8I-M). PSMG3 expression was predominantly enriched in the epithelial cell cluster and was significantly elevated in TNBC epithelial cells compared to normal cells (*p* < 2.2x10^-16^) (Figure 8N). Expression profiles of other *PSMG* family genes at the single-cell level are detailed Supplementary Figure S14.

### Drug Sensitivity Prediction and Molecular Docking

To identify potential therapeutic agents targeting PSMG3-overexpressing tumors, we performed a comprehensive drug sensitivity analysis using the GDSC and CTRP databases. We specifically screened for compounds exhibiting a significant negative correlation between *PSMG3* mRNA expression and the half-maximal inhibitory concentration (IC_50_), where a negative correlation indicates that high PSMG3 expression renders cancer cells more sensitive to the drug. In the GDSC dataset (Figure 9A), we identified several small-molecule inhibitors with significant negative correlations, prominently featuring the EGFR/HER2 inhibitors lapatinib, afatinib, and gefitinib. Similarly, analysis of the CTRP dataset (Figure 9B) highlighted tanespimycin (17-AAG), an Hsp90 inhibitor, as a top candidate with a robust negative correlation. From these candidates, we prioritized lapatinib, afatinib, and tanespimycin for further validation based on their clinical utility and direct biological relevance to our pathway analysis findings. Lapatinib and afatinib were selected due to their direct inhibition of the ErbB (HER2/EGFR) signaling axis, which aligns with our functional enrichment analysis that identified the "Mitogenic action of ErbB2" as a key signaling pathway significantly associated with PSMG3 expression 79, 80. Additionally, tanespimycin (17AAG) was selected for its ability to inhibit Hsp90, a molecular chaperone essential for the conformational stability of HER2 proteins 81. Given that *PSMG* family genes function as assembly chaperones, targeting the protein stability machinery via Hsp90 represents a synergistic therapeutic strategy to disrupt the PSMG3-driven tumorigenic state, suggesting these correlations of PSMG3 overexpression may engender a specific dependency on the ErbB signaling network and proteostatic mechanisms, creating a therapeutic vulnerability exploitable by these targeted agents.

To validate these pharmacological associations and assess the potential for direct physical interaction, we performed molecular docking simulations between the PSMG3 protein structure (PDB ID: 2Z5E) and the selected compounds. We specifically prioritized lapatinib and tanespimycin for structural validation based on their negative correlations in the drug sensitivity screening and their direct biological relevance to our multi-omics findings. Lapatinib was selected due to its direct inhibition of the ErbB (HER2/EGFR) signaling axis, a pathway that aligns with our functional enrichment analysis which identified the "Mitogenic action of ErbB2 in breast cancer" as a key signaling pathway significantly associated with PSMG3 expression. Concurrently, tanespimycin was selected for its ability to inhibit Hsp90, a molecular chaperone essential for stabilizing HER2 proteins. Given that *PSMG* family genes themselves function as assembly chaperones for the proteasome, targeting the protein stability machinery via Hsp90 represents a synergistic therapeutic strategy to potentially disrupt the PSMG3-driven tumorigenic state. The 3D molecular docking visualizations (Figure 9C-E) demonstrated that all selected drugs fit within the binding pocket of PSMG3, exhibiting favorable spatial complementarity. Detailed 2D interaction diagrams (Figure 9F-H) further elucidated the binding stability mechanisms. Specifically, the tanespimycin complex demonstrated a binding energy of -5.4 kcal/mol, as shown in the 3D visualization, with key interactions detailed in the 2D diagram (Figure 9D). The lapatinib complex (Figure 9E) demonstrated a binding energy of -7.1 kcal/mol*.* The detailed 2D interaction diagrams (Figure 9F) further elucidated the binding stability mechanisms of lapatinib, which involved multiple stabilizing forces including Van der Waals, conventional hydrogen bonds, Pi-cation, and Pi-alkyl interactions. These docking results structurally corroborate the drug sensitivity data, suggesting that PSMG3 possesses a druggable pocket that can be effectively targeted by agents like lapatinib and tanespimycin.

## Discussion

The ubiquitin-proteasome system (UPS) is a central regulator of intracellular protein homeostasis, and its dysregulation is a well-established hallmark of cancer development and progression 82. Although pharmacological inhibition of the 20S proteasome core has achieved clinical success in hematologic malignancies, the biological and clinical significance of proteasome assembly chaperones (PACs) in solid tumors remains largely unexplored 17, 83, 84. In this study, we applied an integrated multi-omics framework encompassing bulk and single-cell transcriptomics, epigenetic profiling, immune deconvolution, and molecular docking to systematically characterize the *PSMG* gene family in breast cancer. Our analyses identified PSMG3 as a robust prognostic biomarker and a potential therapeutic vulnerability, particularly in the aggressive TNBC subtype. Among all PSMG family members, PSMG3 demonstrated the most consistent and reproducible dysregulation across datasets. It was significantly elevated in breast cancer tissues relative to normal controls in TCGA-BRCA cohort, a finding independently validated in three external GEO datasets (GSE42568, GSE21422, and GSE61304). Clinically, elevated PSMG3 expression was independently associated with worse OS in both TCGA and METABRIC cohorts. In contrast, PSMG2 exhibited a protective association, underscoring that despite their cooperative roles in proteasome assembly 85, PAC family members may exert distinct and non-redundant functions in tumor biology. The association between PSMG3 expression and the TNBC subtype, an entity lacking effective targeted therapies and largely dependent on chemotherapy, further emphasizes its clinical relevance as a biomarker for high-risk disease. *PSMG3* was among the most significantly elevated genes in TCGA-BRCA, displayed distinct promoter methylation patterns, and showed the significant and most consistent association with adverse survival outcomes across both TCGA and METABRIC cohorts. Notably, PSMG3 was overexpressed in aggressive breast cancer subtypes, including TNBC, which is characterized by a high proliferative capacity, metabolic rewiring, and poor prognoses 86.

A central conceptual question addressed by this study is whether PSMG3 functions merely as a passive marker of high tumor proliferation or plays an active regulatory role in tumor progression. Our integrated analyses support the latter. First, pathway enrichment analyses revealed specific activation of the “Mitogenic action of ErbB2” pathway and the Hsp90 chaperone network in PSMG3-high tumors, suggesting that PSMG3 contributes to maintaining the stability of oncogenic signaling proteins under conditions of proteotoxic stress. Second, PSMG3-high tumors exhibited a distinct immune-excluded phenotype, characterized by enrichment of Tregs and reduced infiltration of cytotoxic CD8⁺ T cells, implying that PSMG3 may actively shape the TME, potentially through metabolic competition or stress adaptation mechanisms. Third, drug sensitivity analyses revealed synthetic lethality between PSMG3 expression and inhibitors targeting ErbB2 signaling and proteostasis pathways, indicating a specific tumor dependency on PSMG3-mediated proteasome assembly. Supporting this functional role, enrichment analyses consistently linked PSMG3 expression to metabolic reprogramming, particularly OXPHOS, electron transport chain activity, and FA metabolism. While aerobic glycolysis has long been considered a defining feature of cancer metabolism, accumulating evidence suggests that aggressive, chemoresistant, and stem-like TNBC cells preferentially rely on OXPHOS to meet elevated bioenergetic demands 87. In parallel, PSMG3 expression was correlated with hypoxia-associated transcriptional programs. Hypoxia is a potent inducer of the epithelial-to-mesenchymal transition (EMT), promoting metastatic dissemination and therapy resistance. We therefore hypothesized that PSMG3 facilitates tumor adaptation to hypoxic and nutrient-deprived conditions, potentially by enhancing proteasomal degradation of metabolic regulators or hypoxia-inducible factors, thereby sustaining cellular fitness under environmental stress. This metabolic and proteostatic adaptation appears to extend to immune regulation within the TME. A CIBERSORT analysis revealed that PSMG3-high tumors were enriched in immunosuppressive Tregs and macrophage populations, while a TIMER analysis demonstrated a broad negative correlation between PSMG3 expression and infiltration of cytotoxic immune effectors, including CD8⁺ T cells and dendritic cells. The coexistence of high Treg abundances with reduced overall immune infiltration is indicative of an immune-excluded tumor phenotype, in which immune cells are prevented from effectively penetrating the tumor core. Such an immunosuppressive milieu likely contributes to the poor survival outcomes observed in patients with high PSMG3 expression. Importantly, these bulk transcriptomic findings were corroborated at single-cell resolution. An scRNA-seq analysis demonstrated that PSMG3 expression is predominantly confined to the epithelial (tumor) compartment rather than stromal or immune cells, and is significantly elevated in TNBC epithelial cells compared to normal breast epithelial cells. Moreover, drug sensitivity screening revealed that PSMG3-high tumors exhibit increased sensitivity to lapatinib, a dual EGFR/HER2 inhibitor, and tanespimycin, an Hsp90 inhibitor 79, 80. This observation aligns closely with our pathway enrichment results highlighting ErbB2 signaling. Mechanistically, this suggests a synergistic vulnerability: PSMG3 supports proteasome assembly and protein turnover, while Hsp90 stabilizes oncogenic drivers such as HER2 88. We propose that PSMG3-overexpressing tumors are hyper-dependent on this interconnected chaperone network to manage proteotoxic stress and sustain oncogenic signaling, rendering them particularly susceptible to combined proteostasis and ErbB2 pathway inhibition.

Despite the comprehensive and integrative multi-omics approach employed in this study, several limitations should be acknowledged. First, although we identified an association between PSMG3 overexpression and coordinated metabolic and immune programs, these findings should be interpreted with caution, as aggressive breast cancer subtypes, particularly TNBC, are intrinsically characterized by high proliferation rates and elevated metabolic activity. Consequently, some observed features, such as enrichment of OXPHOS signatures or immune-excluded phenotypes, may partially reflect general tumor aggressiveness rather than PSMG3-specific regulatory effects. Second, this study is entirely computational and based on retrospective public datasets; thus, causal relationships between PSMG3 and downstream pathways, including OXPHOS and ErbB2 signaling, cannot be definitively established from correlation and enrichment analyses alone. Experimental validation using targeted genetic perturbation and functional metabolic or signaling assays are required to confirm these mechanisms. Third, although PSMG3 retained independent prognostic significance after adjusting for clinical covariates, time-dependent receiver operating characteristic (ROC) analyses yielded only moderate predictive performance, indicating that PSMG3 may be insufficient as a standalone biomarker and would likely be more informative when incorporated into a composite immune-metabolic prognostic model. Finally, cohort heterogeneity and potential confounding factors, such as tumor purity, the molecular subtype, and proliferation status, may have influenced the observed associations, underscoring the need for prospective validation in well-annotated clinical cohorts to fully establish the translational utility of PSMG3.

## Conclusions

**Figure 10:**
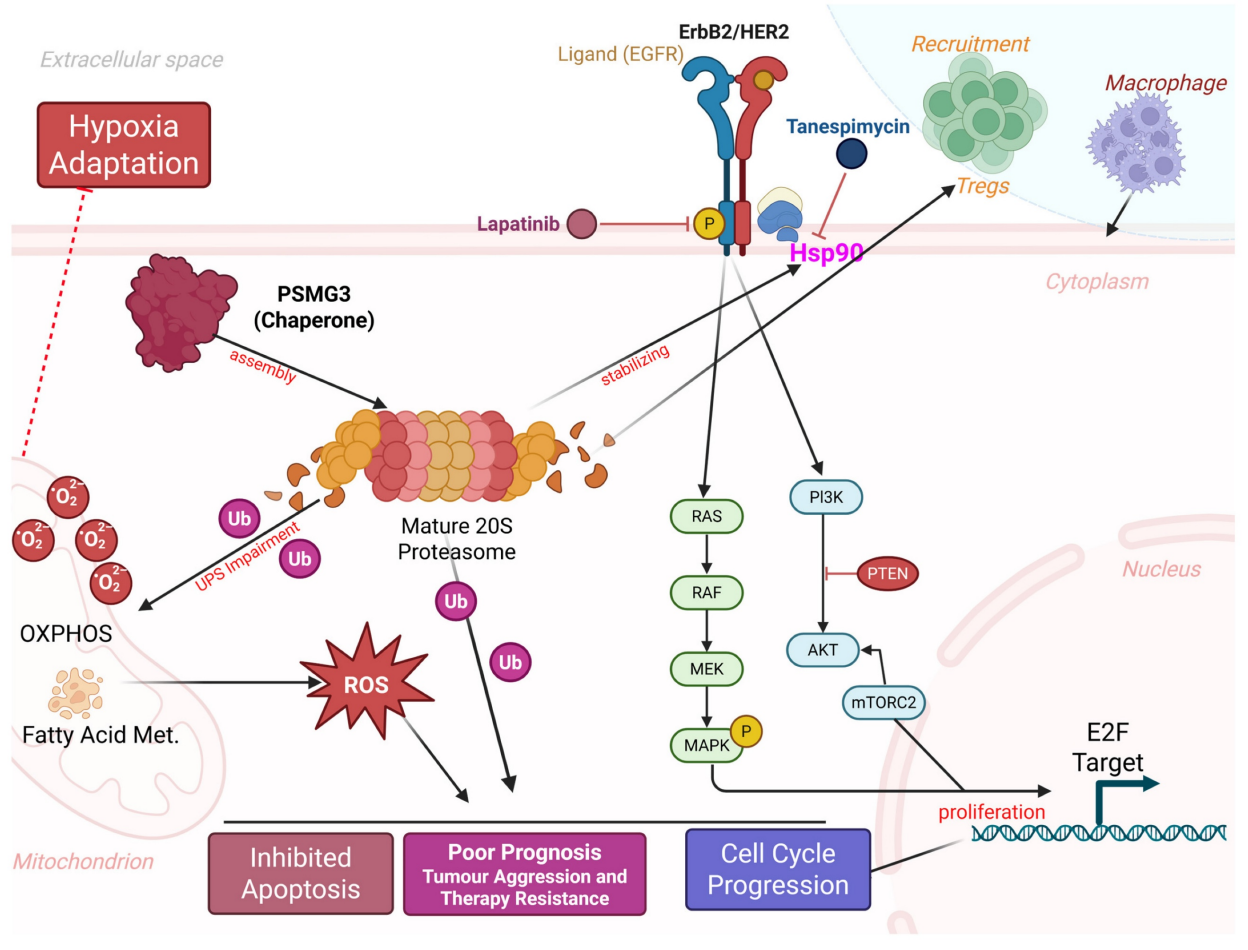
**Proposed model of PSMG3-driven oncogenic signaling and microenvironmental remodeling in breast cancer.** PSMG3 facilitates 20S proteasome assembly and is associated with enhanced EGFR/ErbB2 signaling, promoting downstream PI3K-AKT-mTOR and RAS-RAF-MEK-MAPK activation to support cell-cycle progression and proliferation. Perturbation of proteostasis and increased ubiquitinated protein burden elevate ROS, reinforcing hypoxia adaptation, apoptosis resistance, and therapeutic tolerance, with additional ROS input from mitochondrial OXPHOS and fatty-acid metabolism programs. The model also links PSMG3-high tumors to an immune-suppressive milieu characterized by increased Treg and macrophage recruitment and reduced cytotoxic T-cell infiltration. Candidate therapeutics targeting these nodes (such as lapatinib and tanespimycin/HSP90 inhibition) are indicated.

In conclusion, our multi-omics characterization establishes PSMG3 not merely as a proteasome assembly factor, but as a critical, distinct molecular mediator of tumor aggression, particularly in TNBC. We demonstrated that high PSMG3 expression is independently associated with poor patient survival across multiple cohorts and was validated at the single-cell level to specifically be elevated in the epithelial compartment of TNBC cells. Biologically, PSMG3 appears to operate at the nexus of three key hallmarks: proteostasis, metabolic reprogramming, and immune evasion. We propose that its upregulation facilitates the high bioenergetic demand and stress adaptation required by aggressive tumors, evidenced by the significant enrichment of oxidative phosphorylation and fatty acid metabolism signatures, alongside enhanced adaptation to the hypoxic microenvironment. This metabolic shift profoundly influences the tumor microenvironment, resulting in an "immune-excluded" phenotype characterized by low cytotoxic T cell infiltration and high immunosuppressive regulatory T cells, which drives the poor clinical outcomes observed. Crucially, we identified a therapeutic vulnerability in this PSMG3-driven network. The correlation between PSMG3 expression and sensitivity to lapatinib and tanespimycin which target the ErbB2/HER2 axis and its stabilizing chaperone, Hsp90, uncovers a potent synthetic lethal strategy. This finding is structurally supported by molecular docking, suggesting that PSMG3-overexpressing tumors are hyper-dependent on this interconnected chaperone and signaling network. Collectively, our study redefines PSMG3 as a novel prognostic biomarker and provides a compelling rationale for deploying protein-homeostasis inhibitors in a targeted manner for high-risk breast cancer patients (Figure 10).

## Supplementary Material

Supplementary figures and tables.

## Figures and Tables

**Table 1:** Characteristics of datasets

Dataset	Sample sizes	Platform	Application
TCGA-BRCA	Tumor: 1111 Metastatic: 7 Control: 121	Illumina	Differentially expressed gene analysis, survival analysis Co-expression network
GTEx	Control: 459	Illumina TruSeq	Differentially expressed gene analysis
METABRIC	Tumor: 1390 (only included patient alive or died of disease)	Illumina HT-12 v3 microarray	Survival analysis validation
GSE1456	Tumor: 159	Affymetrix HG-133	Survival analysis validation
GSE42568	Tumor: 104 Control: 17	Affymetrix HG-U133_Plus_2	Differentially expressed gene analysis validation
GSE21422	Tumor: 5 DISC: 9 Control: 5	Affymetrix HG-U133_Plus_2	Differential expressed gene analysis validation
GSE61304	Tumor: 58 Control: 4	Affymetrix HG-U133_Plus_2	Differentially expressed gene analysis validation
GSE161529	Control: 6 (29543 cells) TNBC: 4 (20550 cells)	Illumina NextSeq 500	Single cell validation

## Data Availability

The data are available on request from the corresponding author.

## Abbreviations

PSMG: proteasome assembly chaperone (gene family)
TNBC: triple-negative breast cancer
TCGA: The Cancer Genome Atlas
BRCA: breast cancer
DEGs: differentially expressed genes
GO: gene ontology
GSEA: gene set enrichment analysis
TME: tumor microenvironment
RT-qPCR: real-time quantitative polymerase chain reaction [ RT is usually “reverse-transcription” not “real-time”]
AUC: area under the curve
ROC: receiver operating characteristic
RFS: recurrence-free survival
HR: hazard ratio
Tregs: regulatory T cells
NK: natural killer (cells)
EMT: epithelial-to-mesenchymal transition
OXPHOS: oxidative phosphorylation
Hsp90: heat shock protein 90
IC_50_: half-maximal inhibitory concentration
GDSC: Genomics of Drug Sensitivity in Cancer
CTRP: Cancer Therapeutics Response Portal
UMAP: Uniform Manifold Approximation and Projection
CCLE: Cancer Cell Line Encyclopedia
project): GTEx, Genotype-Tissue Expression
METABRIC: Molecular Taxonomy of Breast Cancer International Consortium
GSVA: gene set variation analysis
scRNA-seq: single-cell RNA sequencing
FDR: false discovery rate
GAPDH: glyceraldehyde-3-phosphate dehydrogenase
DMFS: distant metastasis-free survival
ROS: reactive oxygen species
SDI: sociodemographic index
