# Supplementary material for: Proteasome Assembly Chaperone 3 Defines Metabolic-Immune Programs and Poor Prognosis in Breast Cancer via Multi-Omics Approaches
Source: J Cancer. 2026 Feb 18;17(3):583–603. doi: 10.7150/jca.126116 (PMC13003543; doi:10.7150/jca.126116)
Supplement: Supplementary file 1 — Supplementary figures and tables. [file jcav17p0583s1.pdf]

Supplementary Data

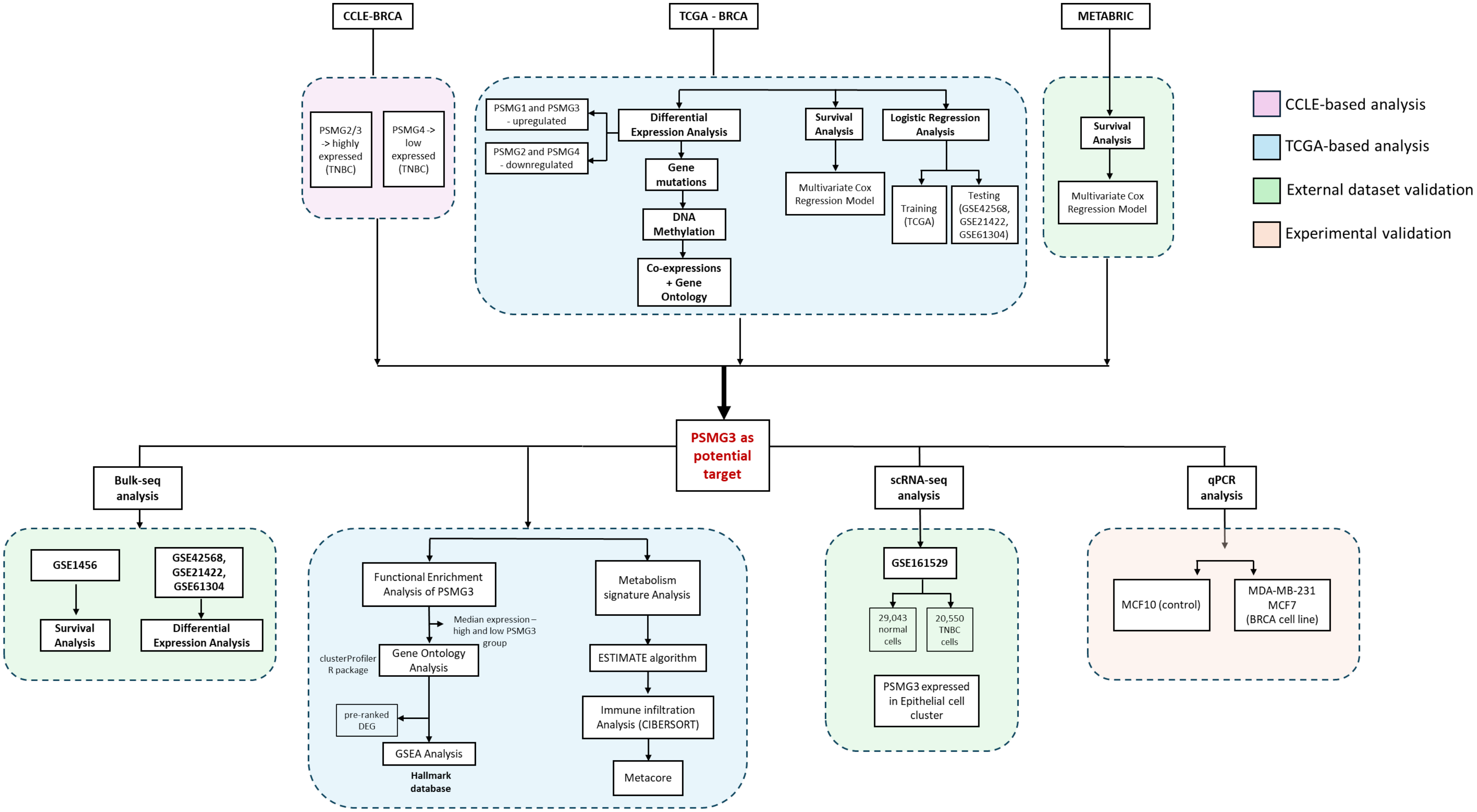

Supplementary Figure S1. Overview workflow of the study

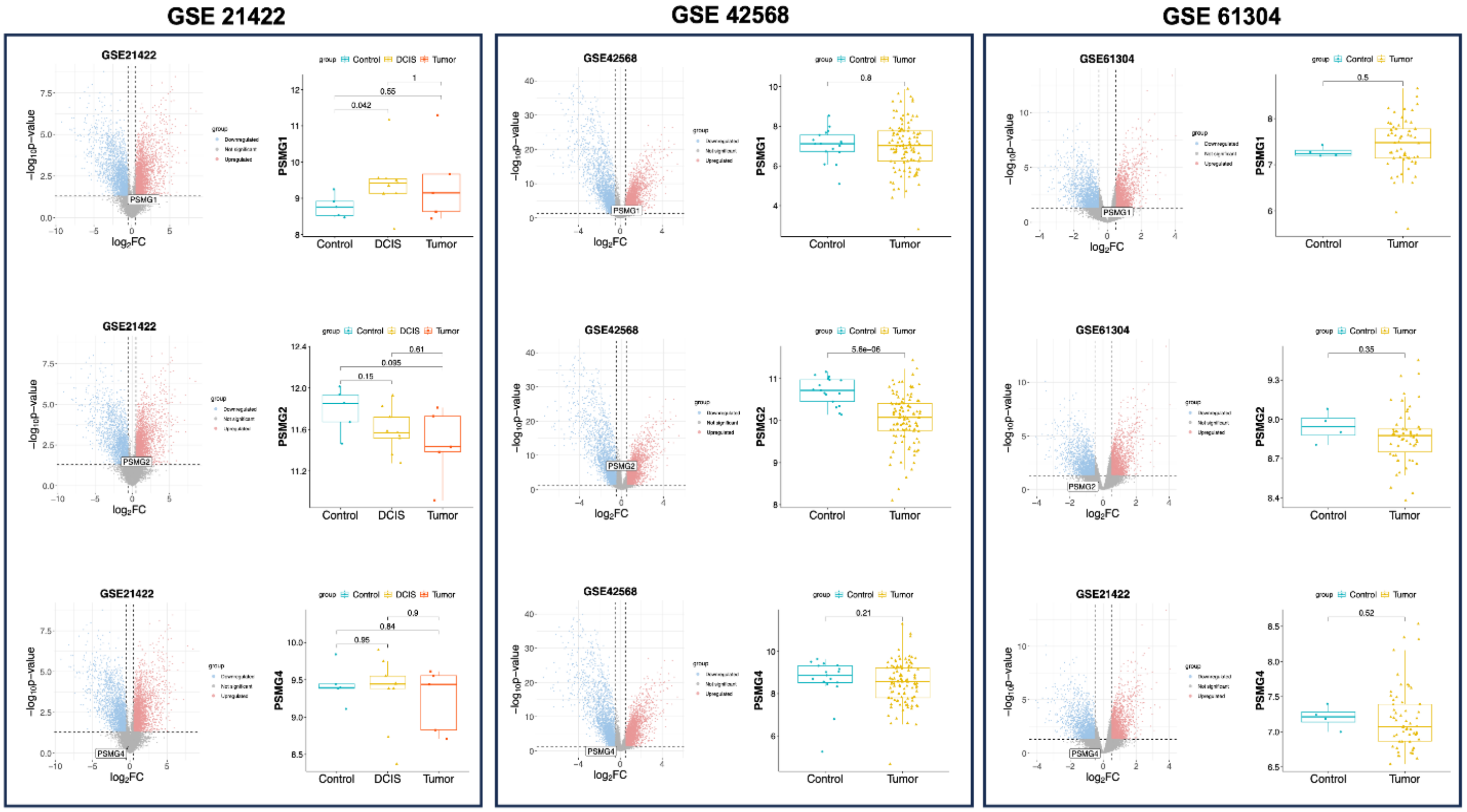

Supplementary Figure S2. Expression analysis of PSMG1, PSMG2, and PSMG4 in external GEO datasets (GSE 21422, GSE 42568, and GSE 61304).

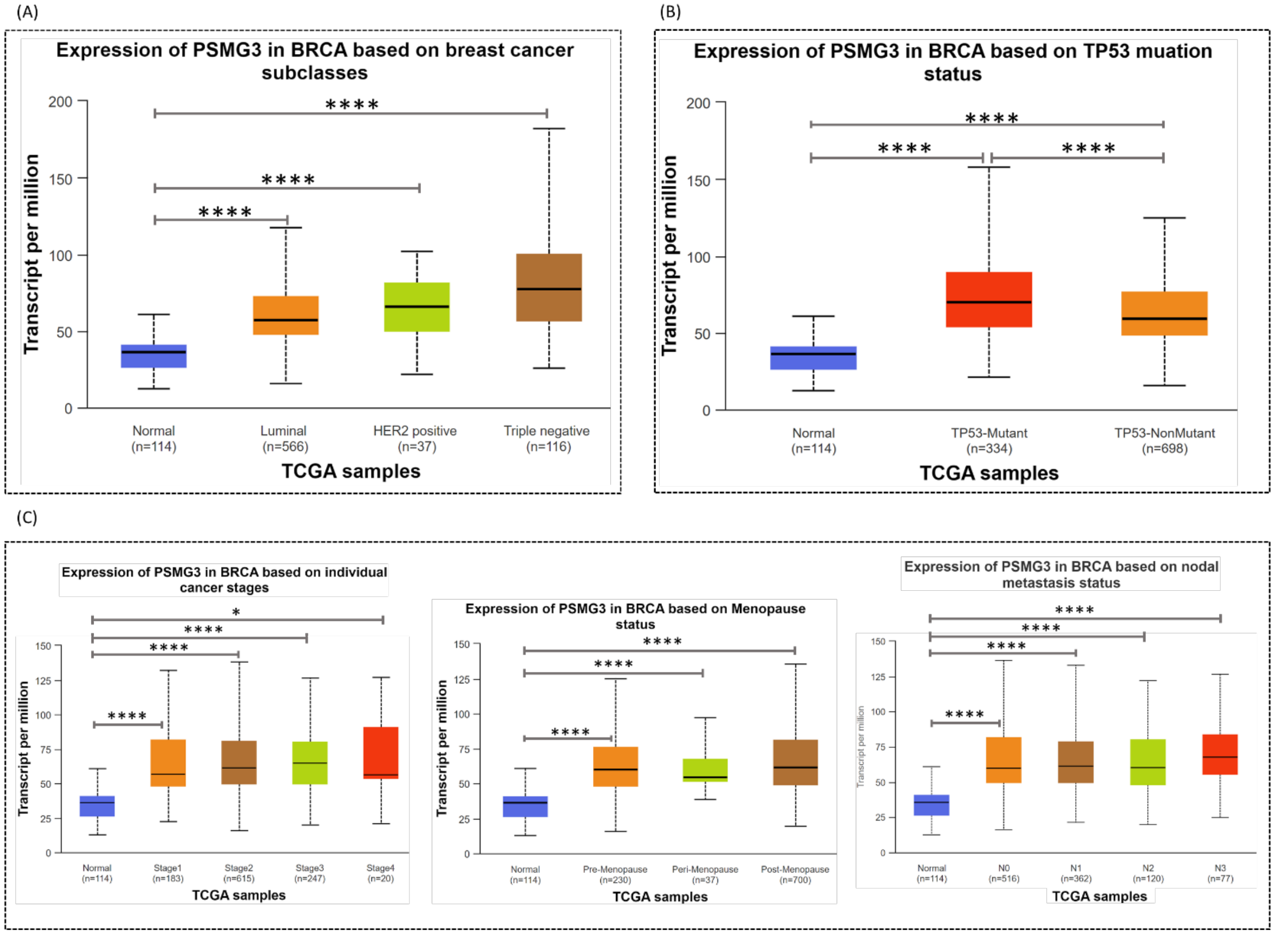

Supplementary Figure S3. Expression level of PSMG3 in TBCA-BRCA dataset. (A) Based on BRCA subclasses; (B) based on the TP53 mutation status; (C) based on individual cancer stages, menopause status, and nodal metastasis status.

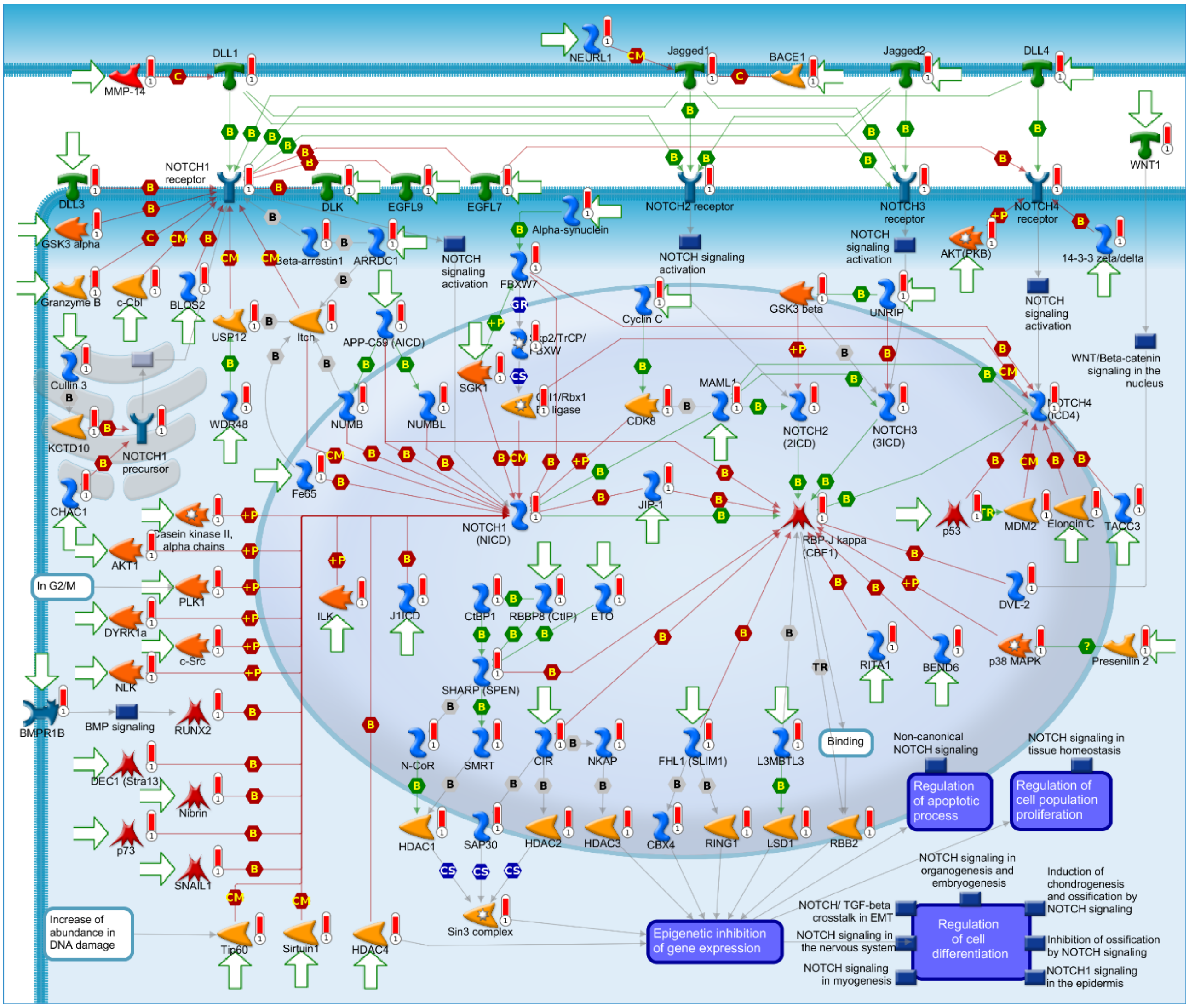

Supplementary Figure S4. Development_NOTCH signaling inhibition: This figure displays the detailed Metacore map for the 2nd pathway, most significantly enriched pathway identified among the 17,238 overlapping co-expressed genes, based on the analysis shown in Figure 6B.

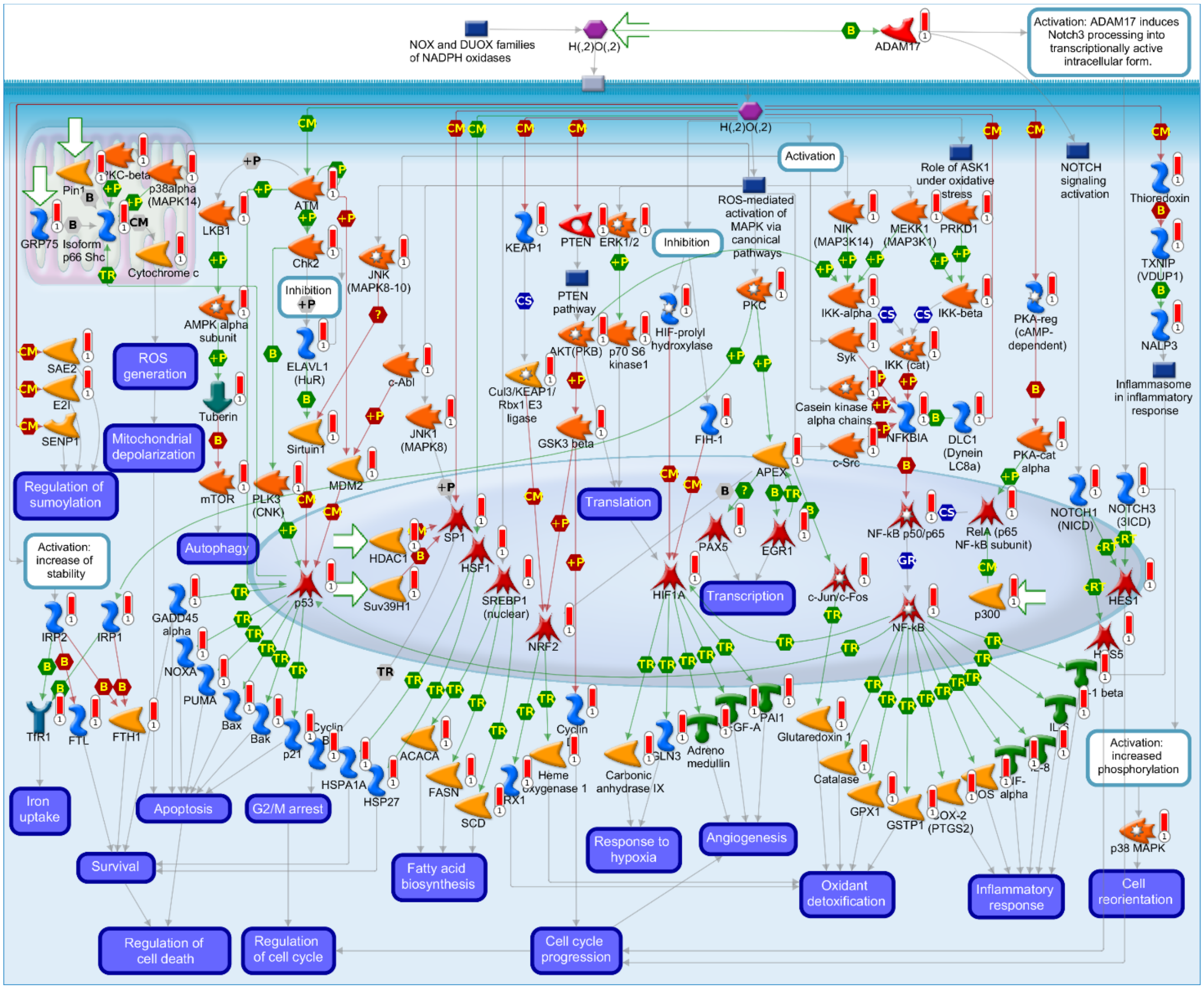

Supplementary Figure S5. Oxidative stress_ROS signaling: This figure displays the detailed Metacore map for the 3rd most significantly enriched pathway identified among the 17,238 overlapping co-expressed genes, based on the analysis shown in Figure 6B.

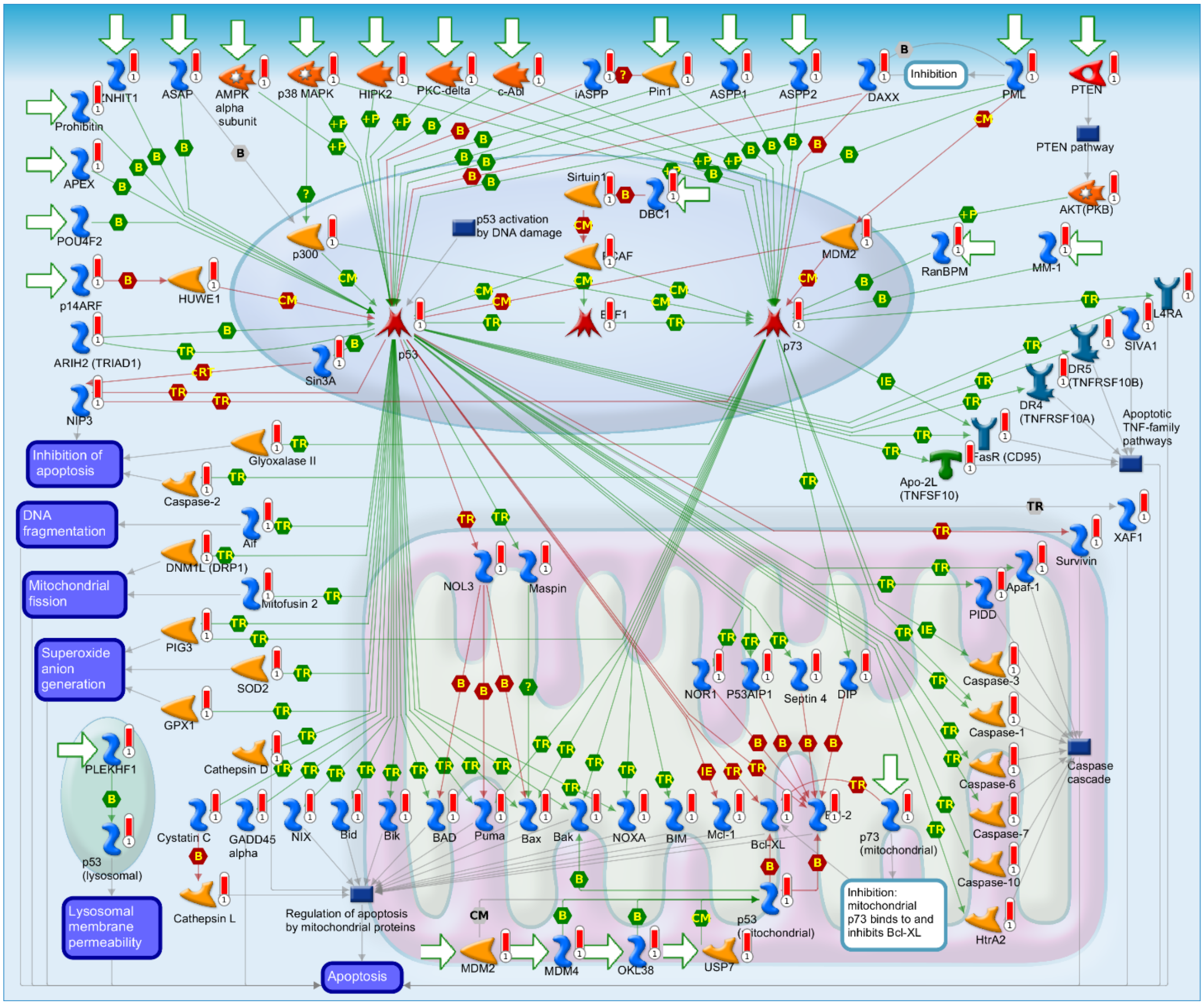

Supplementary Figure S6. Apoptosis and survival_p53 and p73-dependent apoptosis: This figure displays the detailed Metacore map for the 4th most significantly enriched pathway identified among the 17,238 overlapping co-expressed genes, based on the analysis shown in Figure 6B.

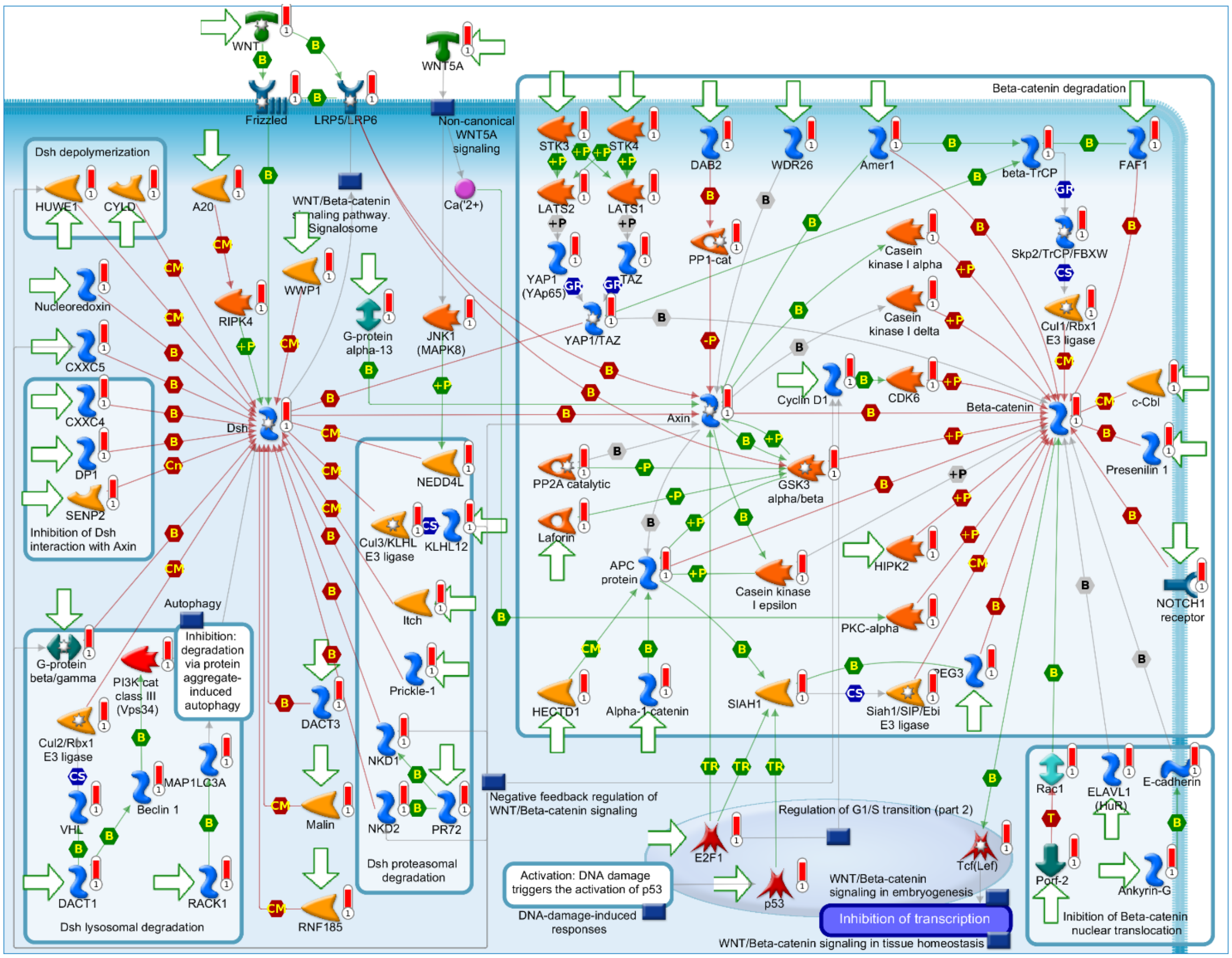

Supplementary Figure S7. Development_Negative regulation of WNTBeta-catenin signaling in the cytoplasm: This figure displays the detailed Metacore map for the 5th most significantly enriched pathway identified among the 17,238 overlapping co-expressed genes, based on the analysis shown in Figure 6B.

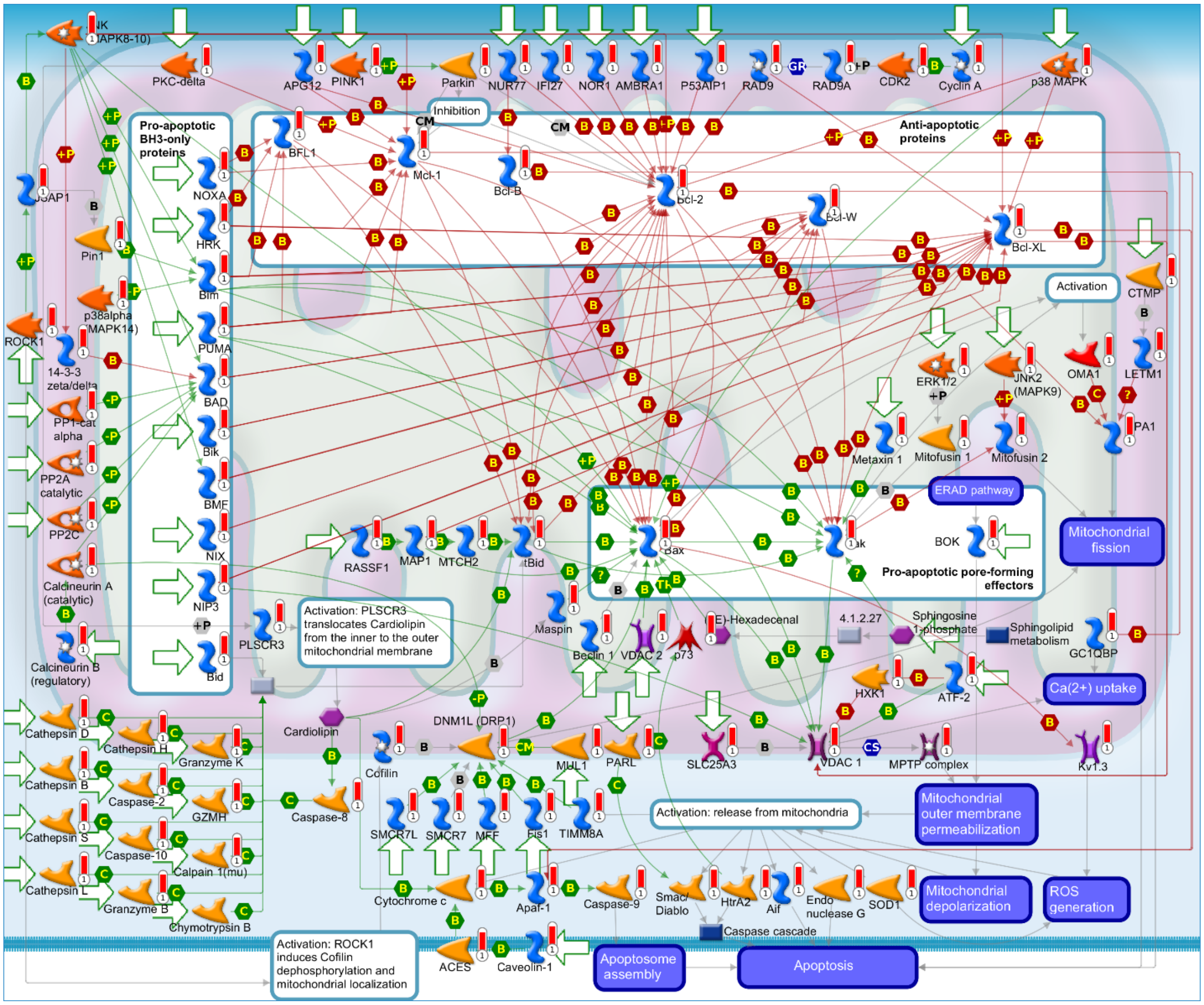

Supplementary Figure S8. Apoptosis and survival_Regulation of apoptosis by mitochondrial proteins: This figure displays the detailed Metacore map for the 6th most significantly enriched pathway identified among the 17,238 overlapping co-expressed genes, based on the analysis shown in Figure 6B.

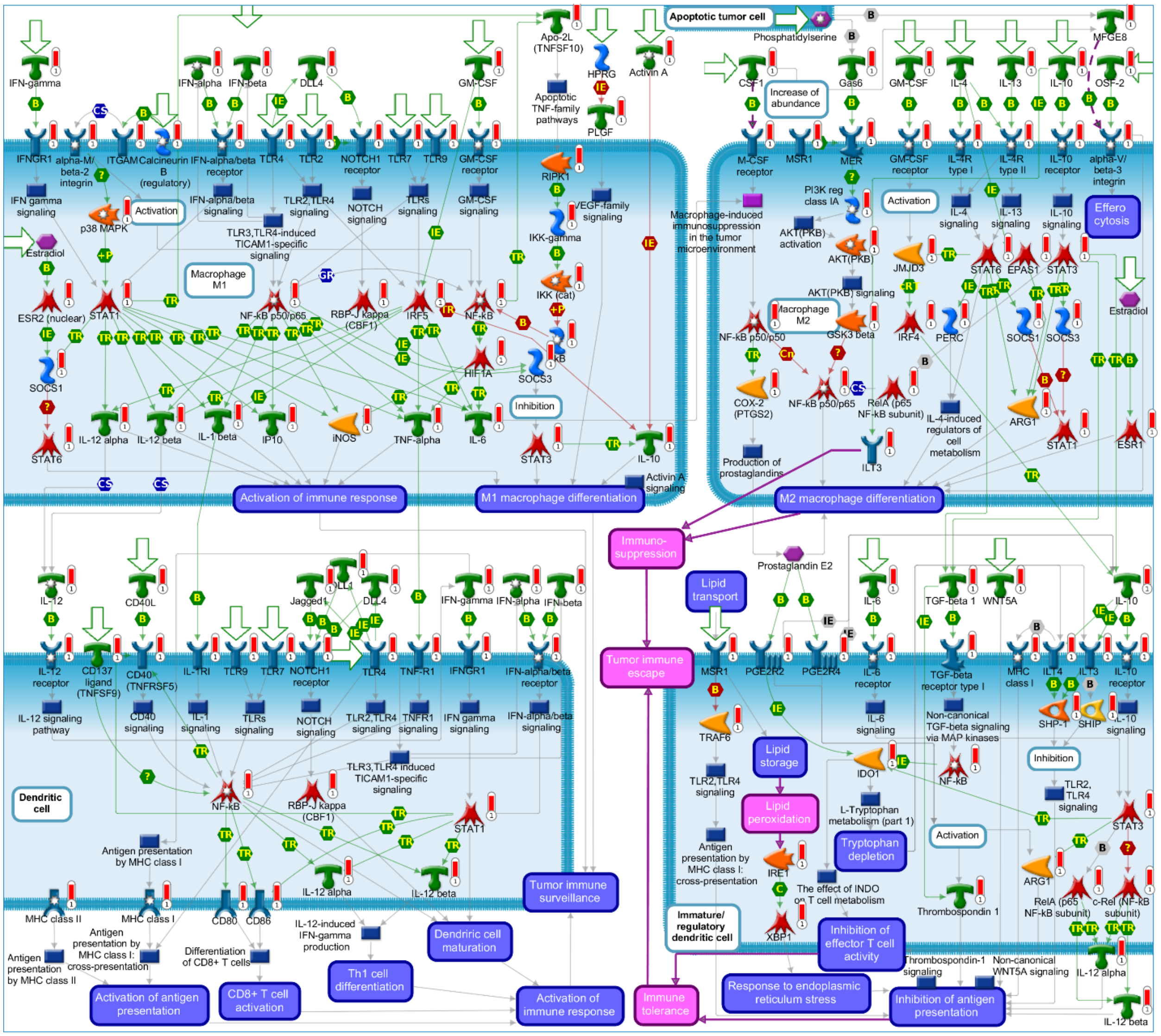

Supplementary Figure S9. Macrophage and dendritic cell phenotype shift in cancer: This figure displays the detailed Metacore map for the 7th most significantly enriched pathway identified among the 17,238 overlapping co-expressed genes, based on the analysis shown in Figure 6B.

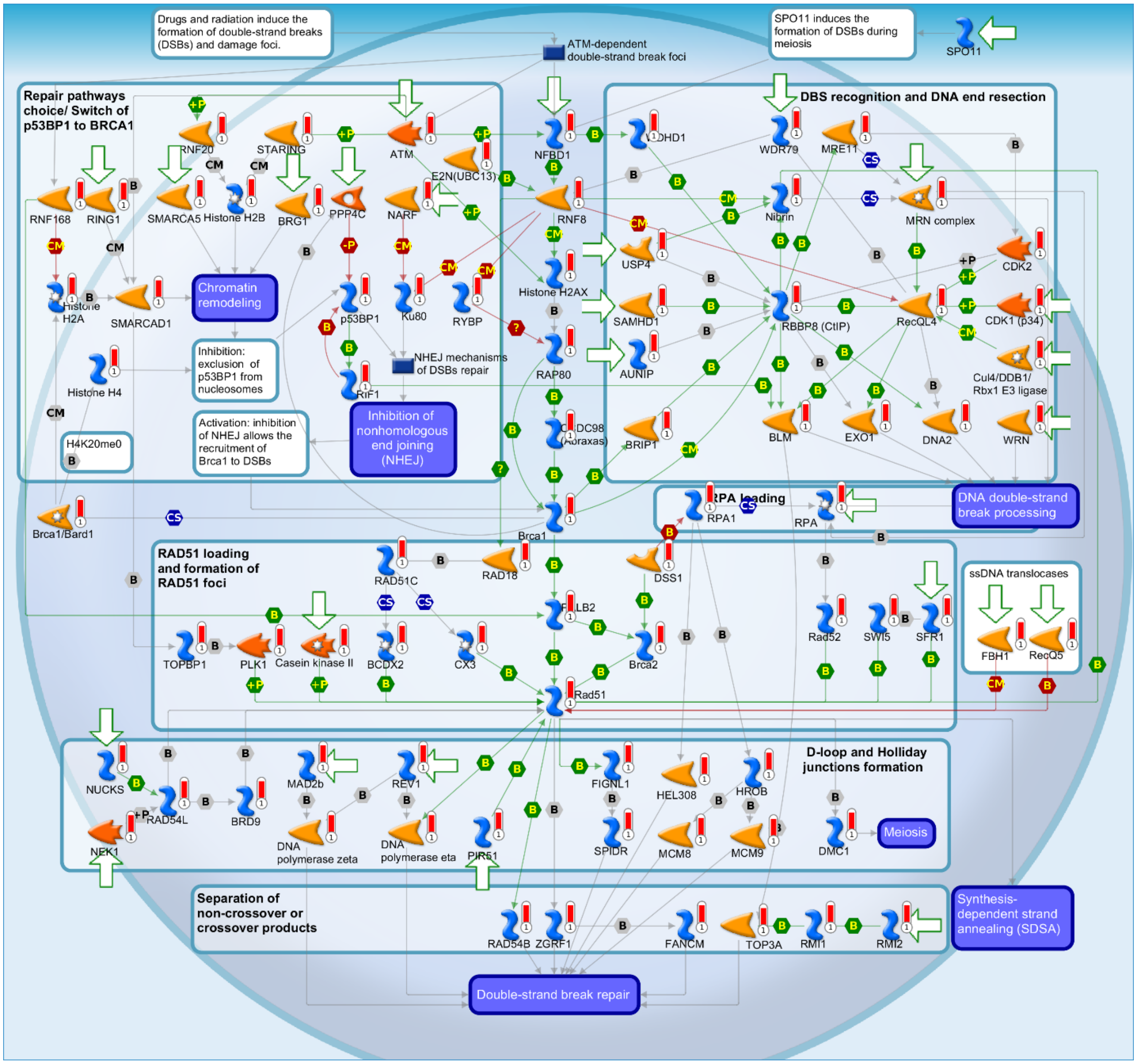

Supplementary Figure S10. DNA damage_Double-strand break repair via homologous recombination: This figure displays the detailed Metacore map for the 8th most significantly enriched pathway identified among the 17,238 overlapping co-expressed genes, based on the analysis shown in Figure 6B.

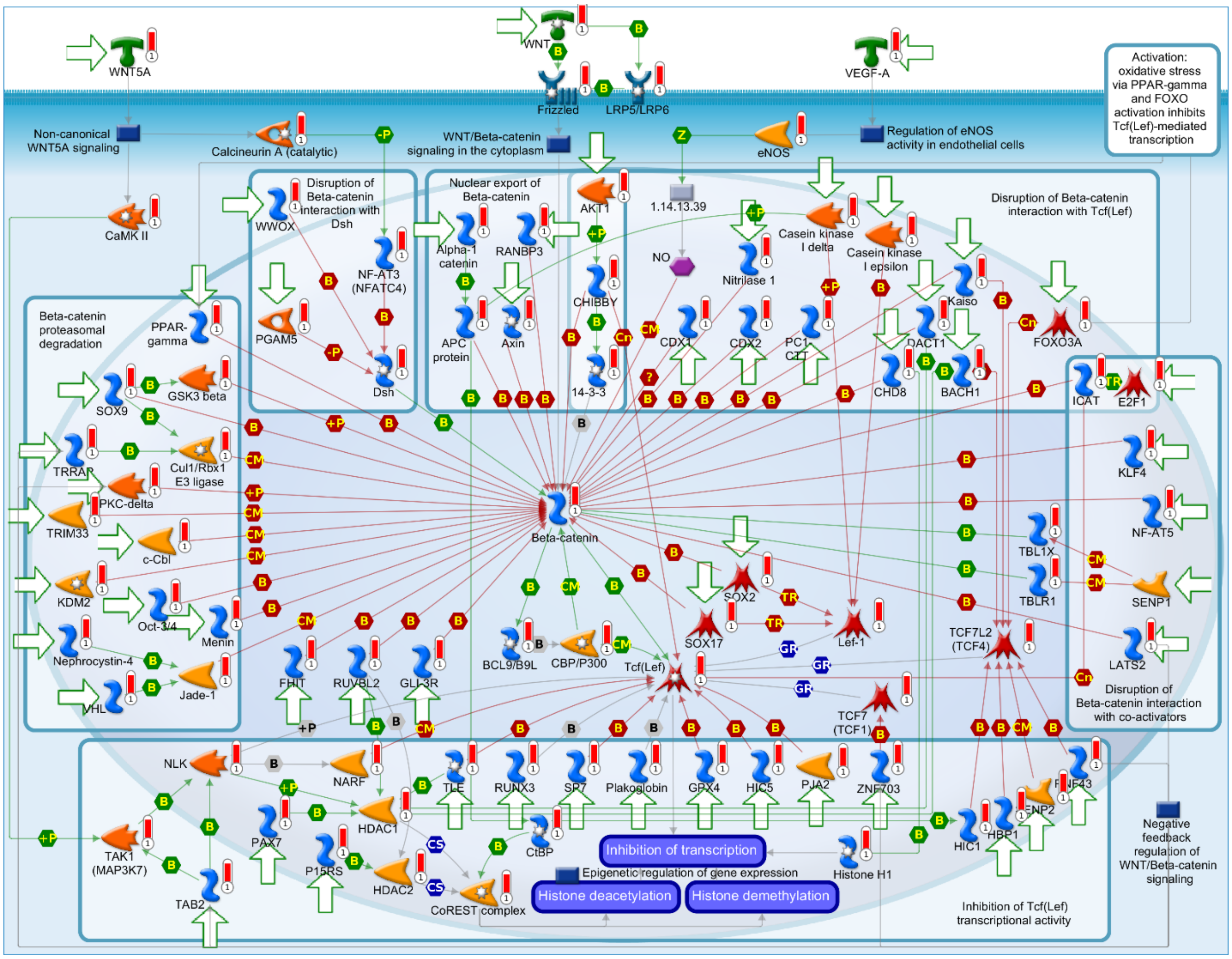

Supplementary Figure S11. Development_Negative regulation of WNTBeta-catenin signaling in the nucleus: This figure displays the detailed Metacore map for the 9th most significantly enriched pathway identified among the 17,238 overlapping co-expressed genes, based on the analysis shown in Figure 6B.

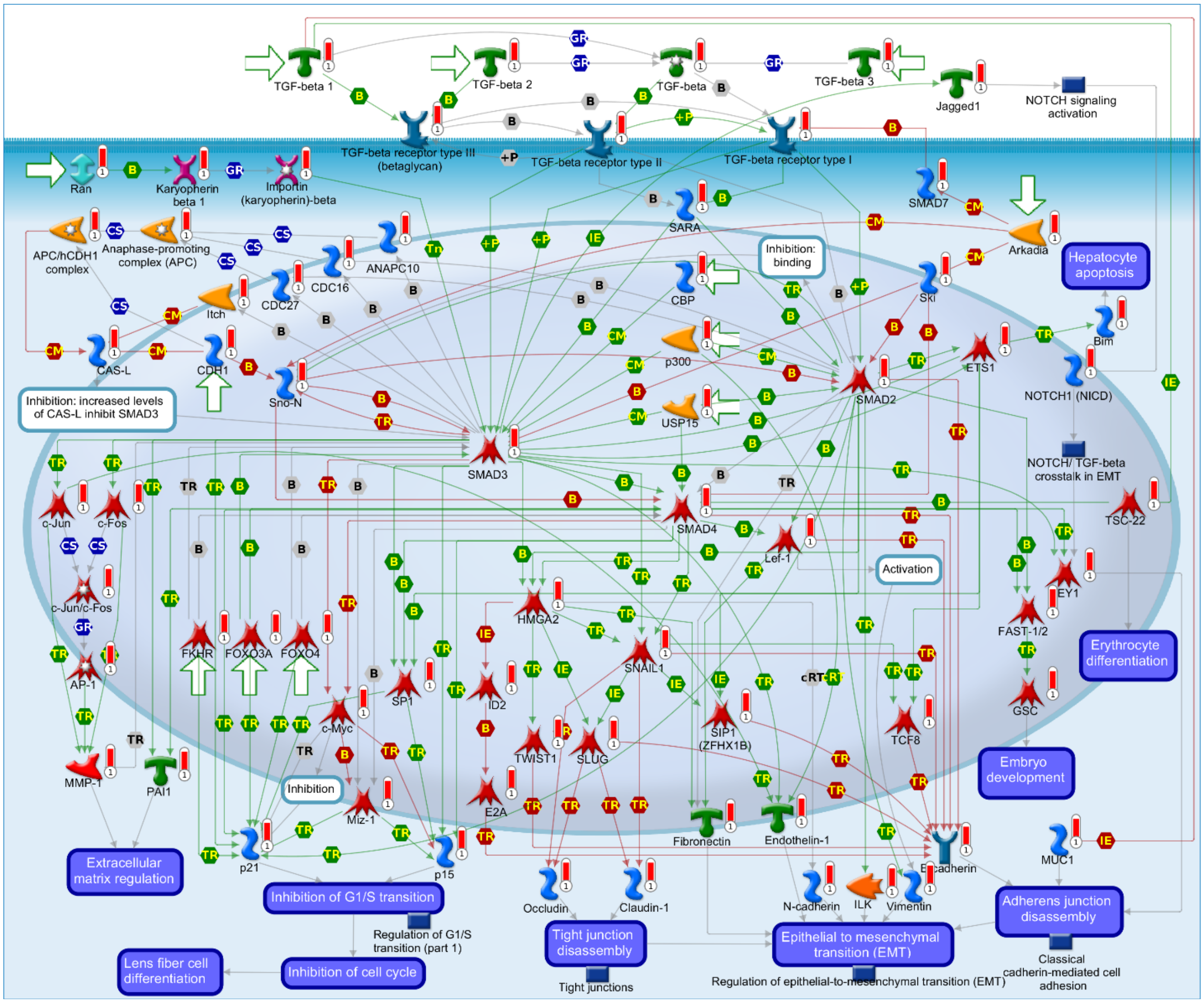

Supplementary Figure S12. Development_Canonical TGF-beta signaling: This figure displays the detailed Metacore map for the 10th most significantly enriched pathway identified among the 17,238 overlapping co-expressed genes, based on the analysis shown in Figure 6B.

GSE1456 (survival analysis)

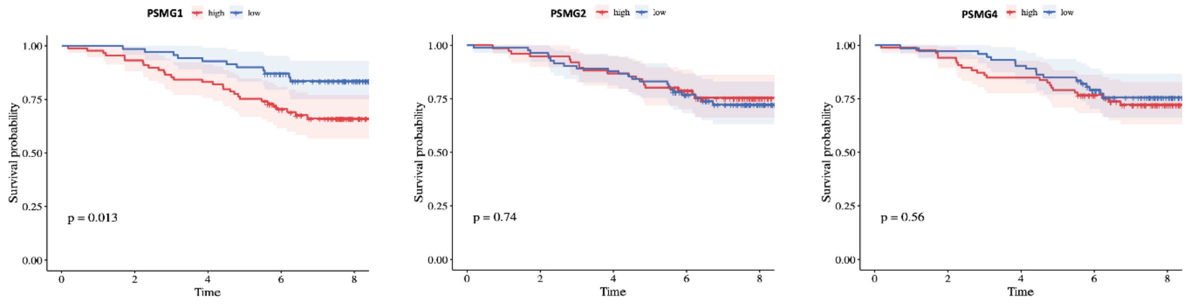

Supplementary Figure S13. Survival analysis of PSMG1, PSMG2, and PSMG4 in the validation GEO dataset (GSE1456).

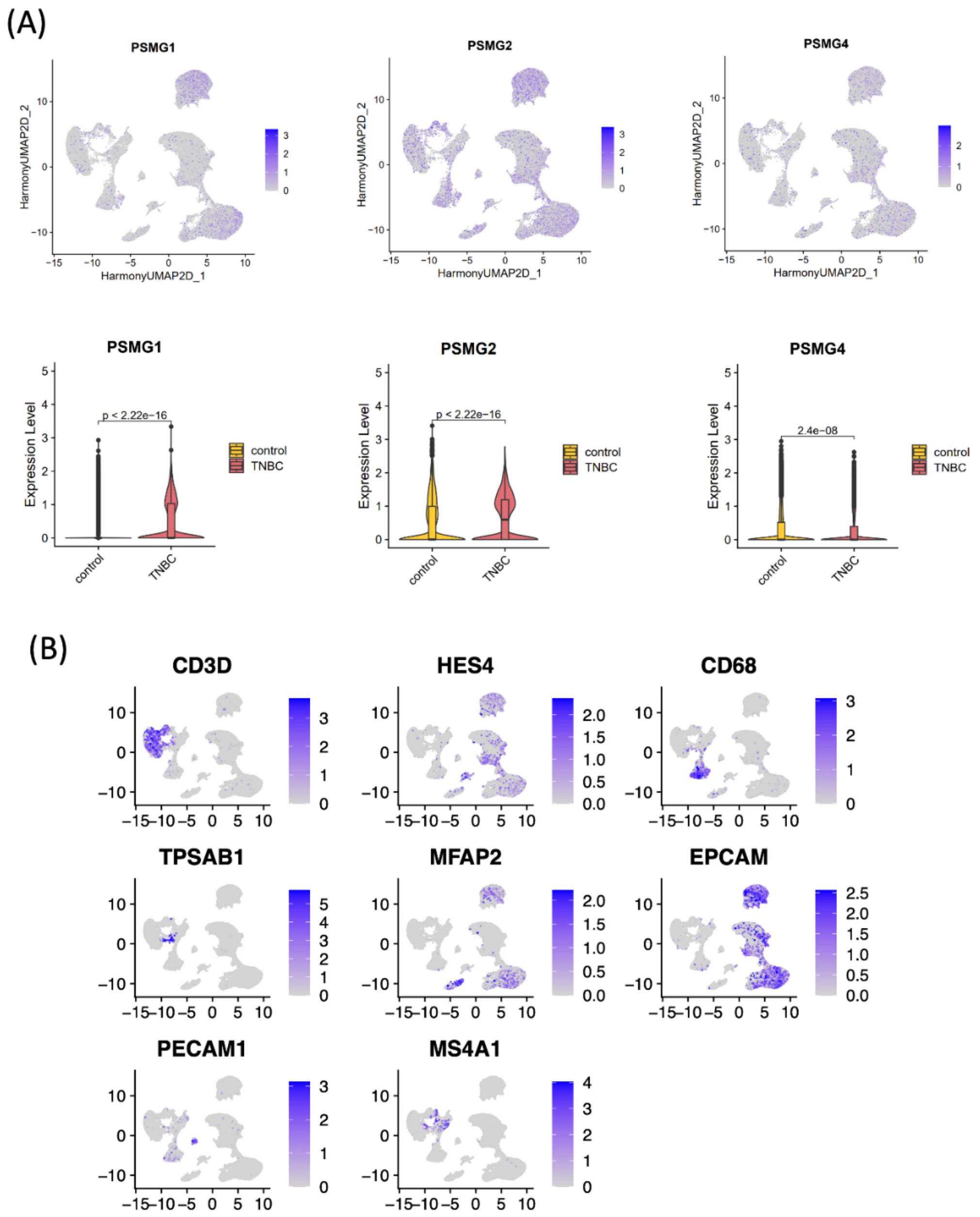

Supplementary Figure S14. **Single-cell RNA-sequencing analysis of PSMG expression and immune cell markers in breast cancer. (A)** UMAP visualization showing the single-cell expression patterns of PSMG1, PSMG2, and PSMG4 across breast cancer samples. Feature plots (top) illustrate the spatial distribution and relative expression levels of each gene, while violin plots (bottom) compare expression between control and triple-negative breast cancer (TNBC) cells. Statistical significance was assessed using the Wilcoxon rank-sum test, with corresponding *p* values indicated. **(B)** UMAP feature plots of canonical cell-type markers used for cell annotation, including CD3D (T cells), HES4, CD68 (macrophages), TPSAB1 (mast cells), MFAP2 (fibroblasts), EPCAM (epithelial cells), PECAM1 (endothelial cells), and MS4A1 (B cells). Color intensity represents normalized gene expression levels.

Supplementary Table 1. Gene Information for Proteasome Assembly Chaperones (PSMGs). This table summarizes key identifiers for the four proteasome assembly chaperones PSMG1 to PSMG4) discussed in the study. Information includes the gene symbol, the full gene description, the corresponding Uniprot ID, and the NCBI Gene ID.

Gene Symbol	**Description**	**Uniprot ID**	**NCBI Gene ID**
PSMG1	Proteasome Assembly Chaperone 1	O95456	8624
PSMG2	Proteasome Assembly Chaperone 2	Q969U7	56984
PSMG3	Proteasome Assembly Chaperone 3	Q9BT73	84262
PSMG4	Proteasome Assembly Chaperone 4	Q5JS54	389362

Supplementary Table 2. Network Objects (Genes) Associated with Top Metacore Enriched Pathways: This table provides the detailed gene lists corresponding to the top enriched Metacore canonical pathways identified in Figure 6B.

S.NO	Maps	pValue	Network Objects from Active Data
1	Transcription_HIF-1 targets	2.269E-27	PDK1, PLGF, P4HA2, PFKL, GLUT3, p21, Carbonic anhydrase IX, PGK1, ID2, F263, Endothelin-1, LOXL4, MMP-2, PLAUR (uPAR), Alpha-1B adrenergic receptor, c-Myc, Mcl-1, Epo, SDF-1, LRP1, ARNT, Heme oxygenase 1, TGF-beta 2, DEC2, ROR-alpha, CITED2, HIF1A, GPI, Endoglin, VEGFR-1, Angiopoietin 2, IBP1, Thrombospondin 1, Mxi1, Carbonic anhydrase XII, ALDOA, NUR77, SOX2, TGF-beta 3, CX3CR1, TGM2, Galectin-1, NOXA, MCT4, ABCG2, p53, MMP-9, PAI1, NIX, TGF-beta 1, PDGF-B, EG-VEGF, Oct-3/4, REDD1, SLC9A1, VEGF-A, Cyclin G2, 5'-NTD, ENO1, MSH6, Stanniocalcin 2, NIP3, MDR1, AK3, IBP3, FECH, Adipophilin, Nucleophosmin, HXK2, WT1, DEC1 (Stra13), ALDOC, CXCR4, TERT, PKM2, G3P2, Ceruloplasmin, iNOS, Lysyl oxidase, Adrenomedullin, HXK1, HIF-1, P4HA1, FGF2, MGF, NANOG, TfR1, GLUT1, MSH2, Transferrin, LOXL2, CTGF, LDHA, HGF receptor (Met), Leptin
2	Development_NOTCH signaling inhibition	5.554E-26	NUMBL, FBXW7, RITA1, SMRT, Granzyme B, DLL3, J1ICD, SGK1, HDAC3, L3MBTL3, NOTCH2 (2ICD), NOTCH4 (ICD4), Tip60, p73, HDAC2, Sin3 complex, BMPR1B, Itch, NOTCH4 receptor, USP12, Fe65, c-Src, PLK1, DVL-2, SHARP (SPEN), Alpha-synuclein, c-Cbl, NKAP, MAML1, 14-3-3 zeta/delta, DYRK1a, CDK8, LSD1, NOTCH2 receptor, EGFL7, Cul1/Rbx1 E3 ligase, ILK, CHAC1, WNT1, BEND6, Sirtuin1, Cyclin C, MMP-14, SNAIL1, HDAC1, SAP30, Beta-arrestin1, p53, CBX4, Casein kinase II, alpha chains, AKT1, NOTCH1 receptor, BACE1, CIR, GSK3 alpha, HDAC4, Jagged2, RBB2, ETO, DLL1, GSK3 beta, TACC3, RING1, MDM2, N-CoR, Skp2/TrCP/FBXW, AKT(PKB), Cullin 3, RBP-J kappa (CBF1), RBBP8 (CtIP), DEC1 (Stra13), Nibrin, JIP-1, NUMB, DLK, NOTCH1 (NICD), BLOS2, Presenilin 2, EGFL9, DLL4, NLK, KCTD10, NOTCH3 (3ICD), FHL1 (SLIM1), RUNX2, NEURL1, Elongin C, NOTCH1 precursor, WDR48, ARRDC1, UNRIP, Jagged1, CtBP1, p38 MAPK, APP-C59 (AICD), NOTCH3 receptor
3	Oxidative stress_ROS signaling	1.913E-24	HES5, SCD, EGR1, IKK (cat), p21, Cytochrome c, E2I, Carbonic anhydrase IX, Bax, PAX5, EGLN3, FTL, IKK-alpha, FTH1, GRP75, Catalase, NF-kB p50/p65, Cyclin B1, c-Src, PUMA, TNF-alpha, Heme oxygenase 1, KEAP1, IRP1, HIF1A, AMPK alpha subunit, Pin1, NIK(MAP3K14), JNK1(MAPK8), GSTP1, COX-2 (PTGS2), ELAVL1 (HuR), PKC-beta,

			Sirtuin1, p70 S6 kinase1, PKC, LKB1, HES1, IKK-beta, NOXA, JNK(MAPK8-10), HIF-prolyl hydroxylase, HDAC1, SP1, p53, NRF2, ADAM17, PAI1, mTOR, Casein kinase II, alpha chains, p38alpha (MAPK14), Tuberin, SREBP1 (nuclear), ERK1/2, c-Jun/c-Fos, VEGF-A, PKA-reg (cAMP-dependent), TXNIP (VDUP1), Bak, FASN, Syk, IL-1 beta, GSK3 beta, IRP2, MDM2, IL-6, GADD45 alpha, NF-kB, FIH-1, AKT(PKB), Suv39H1, NOTCH1 (NICD), Thioredoxin, PLK3 (CNK), Chk2, ACACA, RelA (p65 NF-kB subunit), iNOS, Cul3/KEAP1/Rbx1 E3 ligase, Adrenomedullin, PKA-cat alpha, IL-8, SRX1, Isoform p66 Shc, Glutaredoxin 1, p300, NFKBIA, HSPA1A, TfR1, HSF1, NOTCH3 (3ICD), PRKD1, GPX1, PTEN, MEKK1(MAP3K1), HSP27, ATM, DLC1 (Dynein LC8a), c-Abl, Cyclin D1, SAE2, p38 MAPK, APEX, NALP3
4	Apoptosis and survival_p53 and p73-dependent apoptosis	2.212E-22	Maspin, DR5(TNFRSF10B), p14ARF, PML, Caspase-6, Prohibitin, Apo-2L(TNFSF10), BAD, Cystatin C, ZNHIT1, p73, Bax, Survivin, PIDD, Caspase-1, Mcl-1, PUMA, DBC1, MDM4, Cathepsin L, NOR1, p53 (mitochondrial), PLEKHF1, iASPP, Glyoxalase II, PKC-delta, ASPP2, DAXX, Septin 4, AMPK alpha subunit, Pin1, RanBPM, Sirtuin1, Caspase-10, Caspase-7, Bcl-2, FasR(CD95), NOXA, DNM1L (DRP1), p53, Bim, P53AIP1, NIX, OKL38, SOD2, Apaf-1, ASPP1, Bik, Bak, NIP3, MM-1, DIP, PCAF, ASAP, Caspase-3, MDM2, Caspase-2, Bcl-XL, GADD45 alpha, PIG3, AKT(PKB), DR4(TNFRSF10A), XAF1, Cathepsin D, Aif, Sin3A, HIPK2, ARIH2 (TRIAD1), E2F1, HUWE1, IL4RA, p300, GPX1, PTEN, USP7, c-Abl, SIVA1, HtrA2, NOL3, Mitofusin 2, p38 MAPK, APEX, Bid
5	Development_Negative regulation of WNT/Beta-catenin signaling in the cytoplasm	1.491E-21	CXXC4, DP1, PP1-cat, Alpha-1 catenin, Casein kinase I epsilon, CXXC5, DAB2, Dsh, Casein kinase I alpha, Rac1, YAP1 (YAp65), WWP1, LATS1, STK4, SIAH1, Itch, G-protein beta/gamma, TAZ, Axin, E-cadherin, c-Cbl, STK3, SENP2, JNK1(MAPK8), A20, Cul1/Rbx1 E3 ligase, YAP1/TAZ, MAP1LC3A, ELAVL1 (HuR), PEG3, Malin, Cul3/KLHL E3 ligase, PKC-alpha, Porf-2, Beclin 1, NEDD4L, p53, Siah1/SIP/Ebi E3 ligase, DACT1, Casein kinase I delta, NOTCH1 receptor, VHL, NKD1, RIPK4, APC protein, Presenilin 1, Laforin, FAF1, Beta-catenin, CYLD, PI3K cat class III (Vps34), Nucleoredoxin, G-protein alpha-13, GSK3 alpha/beta, Ankyrin-G, LATS2, WDR26, Skp2/TrCP/FBXW, RACK1, KLHL12, HECTD1, LRP5/LRP6, Prickle-1, HIPK2, Tcf(Lef), E2F1, HUWE1, Amer1, beta-TrCP, PP2A catalytic, PR72, RNF185, CDK6, WNT5A, Cul2/Rbx1 E3 ligase, WNT, Cyclin D1, NKD2, DACT3, Frizzled
6	Apoptosis and survival_Regulation of	1.910E-21	Maspin, MPTP complex, Cathepsin H, Granzyme B, ROCK1, MUL1, BFL1, HRK, RAD9A, Cytochrome c, Caspase-8, OPA1, BAD, p73, Bax, OMA1, SOD1, PINK1, PP1-cat alpha, MTCH2, VDAC 1, Calpain 1(mu), Mcl-1, PUMA, Cathepsin L, NOR1, PKC-delta, Fis1, 14-

	apoptosis by mitochondrial proteins		3-3 zeta/delta, LETM1, MAP1, Pin1, BMF, Cathepsin B, Caspase-10, NUR77, ACES, tBid, Bcl-2, Beclin 1, NOXA, JNK(MAPK8-10), JNK2(MAPK9), DNM1L (DRP1), Calcineurin B (regulatory), Bim, Cofilin, P53AIP1, p38alpha (MAPK14), NIX, Bcl-W, PLSCR3, Calcineurin A (catalytic), Apaf-1, ERK1/2, Parkin, BOK, PP2C, VDAC 2, Bik, Bak, Cathepsin S, MFF, Endonuclease G, NIP3, GZMH, Kv1.3, RASSF1, AMBRA1, Mitofusin 1, Caspase-2, Bcl-XL, Cathepsin D, Granzyme K, IFI27, Aif, SMCR7, GC1QBP, HXK1, PP2A catalytic, PARL, Caspase-9, Metaxin 1, JSAP1, RAD9, CDK2, Bcl-B, CTMP, ATF-2, SLC25A3, TIMM8A, SMCR7L, HtrA2, Cyclin A, Mitofusin 2, Caveolin-1, Smac/Diablo, p38 MAPK, APG12, Bid
7	Macrophage and dendritic cell phenotype shift in cancer	2.467E-21	IDO1, MHC class II, IP10, PLGF, MSR1, XBP1, IFN-gamma, IKK (cat), Gas6, Apo-2L(TNFSF10), IL-12, IL-12 alpha, STAT1, NF-kB p50/p50, IFN-alpha, SOCS3, IL-13, MHC class I, IL-10 receptor, SHIP, TLR9, NF-kB p50/p65, OSF-2, TNF-alpha, PGE2R2, MFGE8, PGE2R4, CD80, IL-12 receptor, Activin A, HIF1A, IL-4R type II, CSF1, Thrombospondin 1, STAT3, COX-2 (PTGS2), TLR4, CD40(TNFRSF5), STAT6, JMJD3, Calcineurin B (regulatory), IL-12 beta, CD86, IL-6 receptor, TGF-beta 1, NOTCH1 receptor, alpha-M/beta-2 integrin, ESR1 (nuclear), c-Rel (NF-kB subunit), IL-4R type I, IL-10, M-CSF receptor, SOCS1, IFN-alpha/beta receptor, ESR2 (nuclear), ILT4, IFN-beta, IL-1 beta, ARG1, I-kB, DLL1, GSK3 beta, PI3K reg class IA, alpha-V/beta-3 integrin, GM-CSF, RIPK1, IL-1RI, IKK-gamma, IL-6, SHP-1, NF-kB, IRE1, AKT(PKB), RBP-J kappa (CBF1), TNF-R1, ILT3, CD40L(TNFSF5), IFNGR1, RelA (p65 NF-kB subunit), iNOS, TRAF6, MER, ITGAM, IRF4, DLL4, WNT5A, GM-CSF receptor, IRF5, EPAS1, PERC, CD137 ligand(TNFSF9), TLR7, Jagged1, p38 MAPK, TLR2, TGF-beta receptor type I
8	DNA damage_Double-strand break repair via homologous recombination	8.485E-21	RIF1, NFBD1, RecQ5, BLM, PALB2, CX3, RNF168, Rad51, p53BP1, FIGNL1, DSS1, TOP3A, BRIP1, Rad52, RYBP, MRN complex, RAD54L, Casein kinase II, RNF8, RecQL4, PLK1, Histone H2A, HROB, DNA2, NARF, SMARCAD1, Histone H2B, FBH1, REV1, NUCKS, Cul4/DDB1/Rbx1 E3 ligase, RAD51C, Ku80, MRE11, CCDC98 (Abraxas), SFR1, RMI1, Brca1/Bard1, DMC1, Histone H4, DNA polymerase zeta, Brca2, WDR79, STARING, MCM8, BRG1, MAD2b, EXO1, E2N(UBC13), TOPBP1, DNA polymerase eta, WRN, CDK1 (p34), WDHD1, SAMHD1, BRD9, RING1, BCDX2, RBBP8 (CtIP), PIR51, Nibrin, RAP80, RAD18, ZGRF1, SPIDR, Brca1, Histone H2AX, RMI2, RPA, HEL308, CDK2, SWI5, AUNIP, RAD54B, ATM, NEK1, MCM9, SMARCA5, RPA1, FANCM, RNF20, USP4
9	Development_Negative regulation of WNT/Beta-	9.982E-21	ZNF703, TBL1X, RUNX3, HBP1, Lef-1, Alpha-1 catenin, Jade-1, Casein kinase I epsilon, NF-AT3(NFATC4), PC1-CTT, TLE, CBP/P300, Dsh, HDAC2, ICAT, RANBP3, CtBP,

	catenin signaling in the nucleus		TBLR1, PJA2, HIC5, PPAR-gamma, Axin, KDM2, WWOX, NARF, c-Cbl, PKC-delta, SOX17, TRIM33, GLI-3R, CHD8, SENP2, Cul1/Rbx1 E3 ligase, RNF43, SOX2, GPX4, HDAC1, CDX1, DACT1, SP7, Casein kinase I delta, AKT1, Calcineurin A (catalytic), BACH1, Oct-3/4, VHL, CoREST complex, APC protein, PGAM5, TCF7L2 (TCF4), 14-3-3, Beta-catenin, PAX7, VEGF-A, BCL9/B9L, Nitrilase 1, KLF4, Menin, NF-AT5, FHIT, CaMK II, GSK3 beta, Nephrocystin-4, HIC1, LATS2, P15RS, TAB2, TAK1(MAP3K7), SOX9, TCF7 (TCF1), LRP5/LRP6, TRRAP, Tcf(Lef), CDX2, E2F1, Kaiso, NLK, WNT5A, WNT, FOXO3A, eNOS, CHIBBY, Plakoglobin, RUVBL2, Frizzled, Histone H1
10	Development_Canonical TGF-beta signaling	4.728E-20	CBP, c-Fos, Lef-1, Sno-N, p21, SMAD2, GSC, Fibronectin, Vimentin, ID2, MUC1, Endothelin-1, Itch, TSC-22, c-Myc, Ran, HEY1, N-cadherin, E-cadherin, TGF-beta 2, ETS1, c-Jun, Karyopherin beta 1, Anaphase-promoting complex (APC), ILK, AP-1, FOXO4, TGF-beta 3, USP15, SLUG, APC/hCDH1 complex, p15, HMGA2, SNAIL1, SMAD3, SP1, Bim, PAI1, Arkadia, TGF-beta 1, E2A, c-Jun/c-Fos, SMAD4, Claudin-1, FKHR, ANAPC10, SIP1 (ZFHX1B), CAS-L, TWIST1, CDC16, NOTCH1 (NICD), Importin (karyopherin)-beta, MMP-1, SMAD7, TGF-beta receptor type II, Ski, CDH1, TGF-beta, TGF-beta receptor type III (betaglycan), p300, SARA, TCF8, CDC27, Miz-1, FOXO3A, Occludin, FAST-1/2, Jagged1, TGF-beta receptor type I
